# Investigation of allele specific expression in various tissues of broiler chickens using the detection tool VADT

**DOI:** 10.1038/s41598-021-83459-8

**Published:** 2021-02-17

**Authors:** M. Joseph Tomlinson, Shawn W. Polson, Jing Qiu, Juniper A. Lake, William Lee, Behnam Abasht

**Affiliations:** 1 Department of Animal and Food Sciences, University of Delaware, 531 South College Ave, Newark, DE 19716 USA; 2 Department of Computer and Information Sciences, University of Delaware, Newark, USA; 3 Department of Biological Sciences, University of Delaware, Newark, USA; 4 Department of Applied Economics and Statistics, University of Delaware, Newark, USA; 5 Center for Bioinformatics and Computational Biology, University of Delaware, Newark, USA; 6 Maple Leaf Farms, Inc., Leesburg, IN 46538 USA

**Keywords:** Genetics, Agricultural genetics

## Abstract

Differential abundance of allelic transcripts in a diploid organism, commonly referred to as allele specific expression (ASE), is a biologically significant phenomenon and can be examined using single nucleotide polymorphisms (SNPs) from RNA-seq. Quantifying ASE aids in our ability to identify and understand *cis*-regulatory mechanisms that influence gene expression, and thereby assist in identifying causal mutations. This study examines ASE in breast muscle, abdominal fat, and liver of commercial broiler chickens using variants called from a large sub-set of the samples (n = 68). ASE analysis was performed using a custom software called VCF ASE Detection Tool (VADT), which detects ASE of biallelic SNPs using a binomial test. On average ~ 174,000 SNPs in each tissue passed our filtering criteria and were considered informative, of which ~ 24,000 (~ 14%) showed ASE. Of all ASE SNPs, only 3.7% exhibited ASE in all three tissues, with ~ 83% showing ASE specific to a single tissue. When ASE genes (genes containing ASE SNPs) were compared between tissues, the overlap among all three tissues increased to 20.1%. Our results indicate that ASE genes show tissue-specific enrichment patterns, but all three tissues showed enrichment for pathways involved in translation.

## Introduction

Allele specific expression (ASE) refers to greater abundance of transcripts from one allele in a heterozygous individual when equal levels are expected. The phenomenon is of great interest for identification of *cis*-regulatory mutations or epigenetic modifications that influence gene expression. Detection of ASE is also useful for overcoming some of the limitations of genome-wide association studies (GWAS) and expression quantitative trait loci (eQTL) studies. Functional evaluation of loci identified in GWAS is hampered when variants fall in large linkage disequilibrium (LD) blocks, introns, intergenic regions and gene deserts^1,2^. Also, low statistical power can prevent GWAS from detecting variants with small effect^3,4^. While eQTL studies can help identify putative regulatory functions of variants, many sites maybe incorrectly classified as *cis* or *trans*^5^. Integration of ASE data with GWAS and eQTL studies is an emerging strategy to improve our understanding of the biological mechanisms underlying genetic risk factors for human diseases^6^.

Several studies have explored ASE in chickens, but these have been fairly limited in scope^7–14^. A study by Cheng et al*.* (2015) found that ASE SNPs can be used as markers to select for resistance to Marek’s disease in chickens^7^. Another study examined ASE in the brain and livers of chicken embryos and identified ASE in ~ 17.8% of genes that are expressed in these tissues^13^. However, there is no published information available on ASE in commercial broiler chickens or in economically important tissues such as breast muscle. Therefore, the first objective of the current study is to investigate ASE in three different tissues—liver, fat and breast muscle—from a commercial broiler cross.

One method of detecting ASE uses the binomial test, which was implemented by Degner et al*.* (2009) in the first RNA-Seq ASE analysis^15^. While various software packages for ASE analysis are available and have been comprehensively reviewed by Gu and Wang (2015)^16^, there is currently no software tool to perform ASE detection on a per sample basis from a standard variant call format (VCF) file. The existing bioinformatics pipelines for ASE detection require customized file inputs, which can be difficult to create and often require additional information, such as haplotype blocks, which may be unavailable^17–19^. Therefore, the second objective of this study is to develop custom bioinformatics tools to detect ASE in a streamlined manner using standard input files. In this study we aimed to streamline the entire ASE analysis process into one self-contained user-friendly program that filters a VCF, tests for ASE using the binomial test, controls for the false discovery rate and reports back all the significant findings. We also aimed to create a program where intermediate processing steps were well documented in various output files to ensure overall accuracy of the program. To achieve this second objective, we created a program called VCF ASE Detection Tool (VADT). VADT specifically performs two different types of statistical analysis of the data (varying models) to identify significant ASE results, a meta-analysis (variants) or multi-dimensional p-value adjustment (samples). It should be mentioned we largely focused on the ASE variant results (meta-analysis) for all three tissues and examined the overall biological significance of genes enriched for ASE but did utilize both methods when examining ASE frequency and strength for specific genes of interest.

## Materials and methods

The RNA-sequencing data used in this study was taken from a feed efficiency project that was the topic of previous publications^20,21^ (SRA accession numbers: abdominal fat samples SRP058295 and breast muscle/liver samples SRP255211). A full breakdown of the 68 samples from 3 tissues of 23 chickens utilized in this study can be seen in Supplemental Sect. 1. Figure S1. Liver, abdominal fat and breast muscle samples were obtained from commercial broiler chickens resulting from a 3-way cross of lines B, C and D (B × CD) (Supplemental Sect. 1. Figure S1). All the collected samples came from male chickens to limit sex driven variability in expression. The genetic background of these lines was previously described by Fu et al., 2015 and 2016^22,23^. All of the samples were prepared using Illumina’s TruSeq Stranded mRNA kit and were sequenced using a 75 cycles paired-end sequencing protocol on an Illumina HiSeq 2000 Sequencer. All fastq files were reviewed for sequence quality using FastQC^24^ and all samples passed this quality control step.

### Sequence alignment and variant calling

Sequencing reads were aligned, and variants were called according to GATK’s Best Practices for Calling Variants in RNA-seq^25–28^. This consisted of aligning sequencing reads using STAR (version 2.5.2b) with the two-pass setting^29,30^ to the Gallus_gallus_5.0 genome build (Ensemble release 86)^31,32^. The index file initially used for STAR was created using gtf file 86 from Ensembl^32,33^. It should be mentioned that sequencing reads were not trimmed to avoid potential bias this might introduce^34^, instead soft-clipping was allowed during alignment. After alignment, duplicate reads were marked using Picard (version 2.8.1)^35^. Aligned reads were then submitted to the GATK (version 3.7) pipeline, which includes “Split’N’Trim,” “Base Recalibration” and variant calling using HaplotypeCaller (with the g.vcf setting). The g.vcf files from all the samples listed in Supplemental Sect. 1. Figure S1. along with 32 additional samples from a related project were merged to create a global vcf file. The global vcf file was then used to mask the reference genome using Bedtools^36^. All samples were re-aligned to the masked genome and variants were re-called using all the previously mentioned steps. A detailed summary of this entire process is available in Supplemental Sect. 1. Figure S2. The final g.vcf for the samples was created by merging samples by tissue and using GATK to remove variants with “Fisher Strand > 30.0”, “Quality Depth < 2.0” and “Depth < 100”.

### Examining gene coverage

To verify that no systemic sequencing errors occurred, gene coverage was assessed using RSeQC’s (version 2.6.4) geneBody_coverage.py^37^. The BED file utilized in this analysis was created using the UCSC Genome Browser (Table Browser) utilizing Gallus_gallus-5.0^38^. The outputted BED file was then slightly modified into a format allowable for the program. Analysis was run on the initial BAM file created by Picard when converting SAM to BAM.

### Analyzing unmapped reads

An unexpected discrepancy in alignment rates among tissues warranted further investigation of potential alignment issues. To characterize unmapped reads in a systematic manner, a custom python program called FastqBLAST (https://github.com/juniper-lake/FastqBLAST), was used to remotely submit random samples of sequences to NCBI’s BLAST using the Biopython package^39^. The program takes in a fastq file and randomly selects a sample of sequences based on a user defined parameter. Read ends with a quality score of less than 20 are trimmed until passing bases are identified. If no passing bases are identified the entire sequence is removed. The program then BLASTs the filtered sequences and retrieves the top hit from the BLAST results. Additional gene information is then retrieved using NCBI’s EFetch function and the results are merged. Finally, FastqBLAST tallies all the results and prints out summary reports of the BLAST results and EFetch results. Parameters of FastqBLAST were adjusted to match NCBI’s Megablast parameters. This analysis was conducted on data from a randomly selected chicken that had samples collected from all three tissues with FastqBLAST processing ~ 1000 unmapped sequence reads from each sample.

### Validating RNA-Seq analysis with 600K genotyping data

To validate RNA-seq variant calls, we used 600K genotyping data (GSE131764) obtained using the ThermoFisher Axiom Chicken Genotyping Array^40^. Raw genotyping data (cel files) was analyzed using Axiom Analysis Suite Software (version 3.0.1 64 bit) with the *Gallus gallus* 5.0 genome downloaded from Axiom server following the software’s Best Practices Workflow using recommended settings for agricultural animals^41,42^. Results from this analysis were exported as a VCF file.

The resulting VCF files from the various tissues (RNA-seq) and genotyping panel were compared using a custom python program that filters VCFs and then compares concordance of matching samples and SNPs (https://github.com/mjtiv/Compare_VCF_Files). The 600K genotyping data was filtered to remove all SNPs with < 97% call rates and all non-overlapping samples. The RNA-Seq data was filtered using the following criteria: all SNPs that failed previously mentioned GATK filters, SNPs within 75 base pairs of INDELs, SNPs with < 20 quality score and SNPs with < 20 read counts. Final datasets were then compared for matching SNPs and overlapping SNPs were analyzed for overall concordance among samples.

### VCF ASE detection tool (VADT)

**Figure 1:**
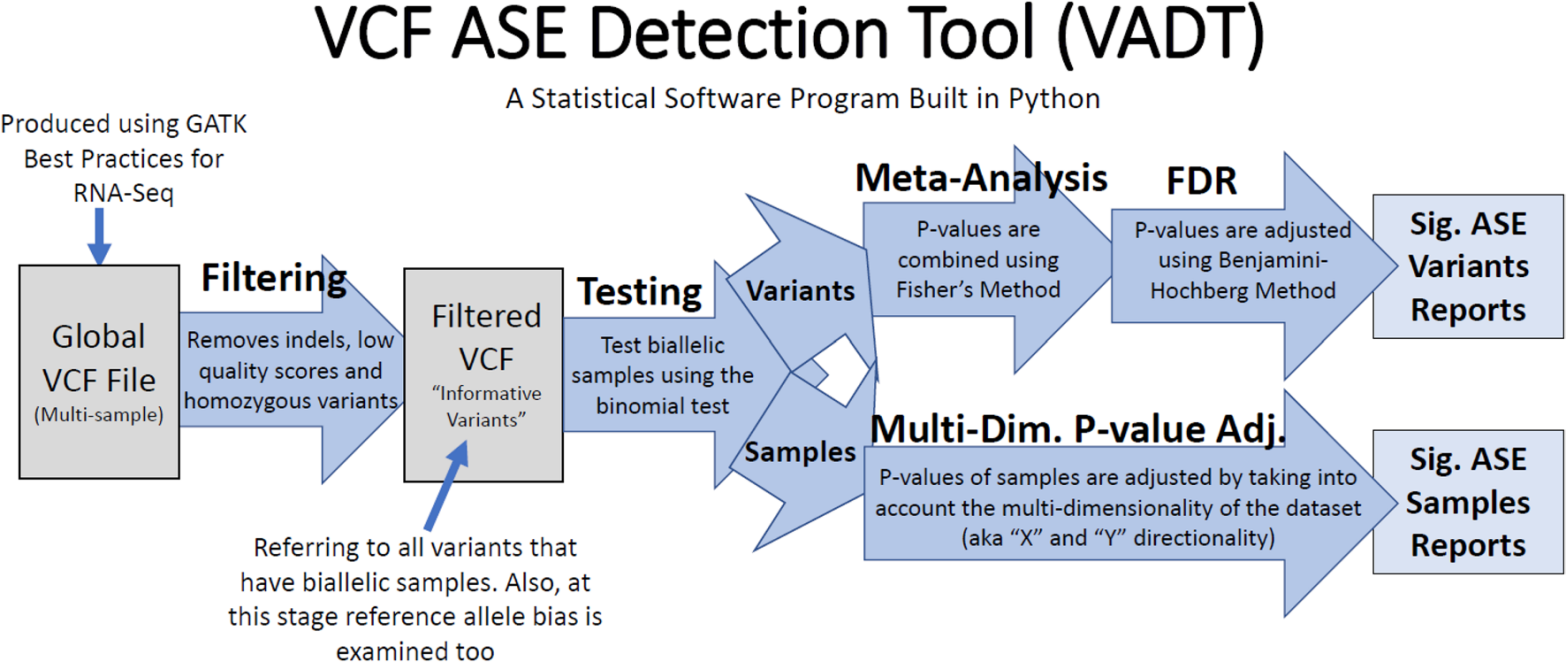
Major steps of the analysis performed by VCF ASE Detection Tool (VADT). The program filters SNPs, tests for ASE and implements one of two methods for multiple testing correction to identify significant ASE variants or samples with significant ASE.

To identify ASE variants, a custom program was developed called VADT (VCF ASE Detection Tool), the complete code for the program can be found at https://github.com/mjtiv/VADT. VADT takes in a raw VCF file, filters the data and then performs various statistical tests for detection of allele specific expression (ASE) to identify highly confident occurrences of ASE. VADT was written in python3.6 and requires NumPY and SciPy^43–45^. A full explanation of the tool and all its possible settings can be found at the prior link and an outline of the major steps of the program seen in Fig. 1.

VADT first filters out variants marked as failing by GATK, indels, SNPs within a certain distance of an indel (user defined), multiallelic SNPs and SNPs with (quality score below < 20). At each remaining SNP, variant calls are removed for samples that are homozygous, have low read count (coverage) or that have a minor allele count below 1% of the total counts.

VADT next performs a binomial test on all remaining SNPs per sample; these testable SNPs will be referred to as “informative SNPs” throughout the paper. To perform the binomial test, VADT utilizes the raw read counts from the VCF file, found in the “genotype fields” under the AD (unfiltered allele depth) sub-field for each sample and implements the test utilizing SciPy’s binomial test^45^. After performing the initial binomial test, two different statistical analyses are implemented to answer different biological questions about the data, and at the same time, control for the false discovery rate (FDR). The two statistical analyses implemented are (i) a meta-analysis to examine variants and (ii) multi-dimensional p-value adjustment to examine both variants and samples. These two statistical analyses are separate from each other and interrogate the data in different ways.

VADT implements a meta-analysis to identify variants that exhibit ASE (i.e., ASE variants) using information from all samples included in the analysis. In the meta-analysis, each SNP’s p-values for testable samples are combined using Fisher's Method^46^. After implementing this procedure on all informative SNPs, the false discovery rate (FDR) is controlled using the Benjamini–Hochberg p-value adjustment method^47^ generating a final adjusted p-value.

The second statistical analysis implemented by VADT examines individual samples to identify samples that exhibit ASE (i.e., ASE samples) for each variant. For ASE sample identification, VADT implements a multi-dimensional FDR-controlling procedure proposed by Guo et al*.* (2010)^48^ and later by Li and Ghosh (2014)^49^ to control mixed directional FDR (mdFDR) or overall FDR (OFDR). This statistical model controls for the type 1 error on the variant levels while allowing one to make inference for individual hypothesis of each variant. A full breakdown of how this method was implemented in VADT can be found in Supplemental Sect. 2.

### VADT settings utilized in study

The present study utilized an indel filter distance of 75 base pairs (read length), a quality score minimum of 20, and a SNP read count threshold of 20 for each sample. The statistical adjustment cutoff utilized by both the meta-analysis and multi-dimensional p-value adjustment was 0.05. However, due to the overall complexity of analyzing both variant and sample data, the focus of this paper will largely be on the biological significance of ASE variants using the meta-analysis results. But we will examine the benefits of utilizing both sets of data when deciphering the biological significance of results regarding ASE frequency, penetrance and strength.

### Biological significance of ASE variants

ASE SNPs identified in this study were submitted to Ensembl’s Variant Effect Predictor (VEP) tool^50^ for functional prediction. The genes associated with these SNPs were also submitted to DAVID for functional annotation^51^ using an adjusted p-value cutoff of 0.1. The overlap of ASE SNPs or genes among tissues was visualized using two online bioinformatics tool for creating Venn diagrams^52,53^.

## Results

### Mapping and initial variant results

RNA sequencing of the 68 samples produced an average of 33,221,292 paired-end reads per sample with an average unique mapping rate of 87.24%. A summary of the mapping results for the samples can be seen in Table 1. Utilizing all 68 samples and an additional 32 samples available in the lab (Supplemental Sect. 1. Table S1), a total of 3,147,284 variants were identified after the first alignment and used to mask the reference genome for the second alignment.

**Table 1:** Summary statistics of STAR alignment (1st pass) for all samples.

	Avg. input reads	Input length	Avg. uniquely mapped reads	% uniquely mapped reads
Breast muscle (n = 23)	34,212,079	150	27,477,523	80.11
Abdominal fat (n = 22)	32,096,465	150	29,137,588	90.78
Liver (n = 23)	33,306,428	150	30,305,272	90.98

### Mapping issue between tissues

The liver and abdominal fat samples had mapping rates ~ 10% higher than the breast muscle samples (Table 1) even though all the samples were prepared using the same methods (the same RNA isolation and cDNA library preparation kits as well as the same Illumina sequencing machine and protocol, see “Methods”). This mapping issue discrepancy could potentially be due to variation in read quality scores, which were originally inspected using the FastQC’s pass/fail categorization and visual inspection of the program’s output report. To use a quantitative approach, a custom program was written to quantify all the FastQC results based on various base quality scores. However, after analysis of all the samples, no issues were found with base quality, and the average base quality per tissue was 34.79 for breast muscle, 34.15 for liver and 36.00 for abdominal fat. To further investigate the mapping issue for systematic bias that could influence gene coverage, the sequencing reads were examined using RSeQC and individual tissues showed no apparent bias in terms of gene coverage (Supplemental Sect. 1. Figures S3).

We eventually determined that breast muscle samples had a significantly higher percentage of reads considered “too short” according to STAR (avg. 15.39% compared to avg. 4.50% for abdominal fat and avg 5.33% for liver with an ANOVA p-value of 9.76 × 10^–24^). STAR describes reads as “too short,” if 2/3 of the read cannot be aligned correctly^29,30^. A sample was randomly chosen for analyzing its unmapped reads files (R1 and R2) from all three tissues using FastqBLAST. A total of 1000 unmapped sequences from each file (Supplemental Sect. 1. Table S2) were randomly selected and BLASTed against the chicken genome (NCBI taxid 9031). The top five genes based on counts of hits were identified for each tissue (Supplemental Sect. 1. Table S3) and we discovered that unmapped reads from muscle samples largely represented muscle-related genes whereas unmapped reads from abdominal fat and liver samples largely represented non-tissue specific genes (i.e., ribosomal and mitochondrial genes). The top 15 gene hits from the unmappable reads for R1 and R2 were verified using Gallus_gallus_5.0 GTF and it was found many of the genes had numerous isoforms that could potentially make alignment problematic (Supplemental Sect. 1. Table S4). Also, it was speculated the latest genome build release could potentially rescue this lower mapping rate, so the prior chosen sample was aligned to the latest genome build (muscle mapping rates old 79.43% vs new 80.92%)^54,55^. A full breakdown of the various tissues can also be seen in Supplemental Sect. 1. Table S5. Also, the prior analysis with GTF files was repeated with the Gallus_gallus_6.0 GTF (Supplemental Sect. 1. Table S4). Overall, it was found the muscle sample using the latest genome build still had the same issue with mapping rate when compared to the other tissues. Also, the results between the two GTF files were very similar for non-mapping genes.

**Figure 2:**
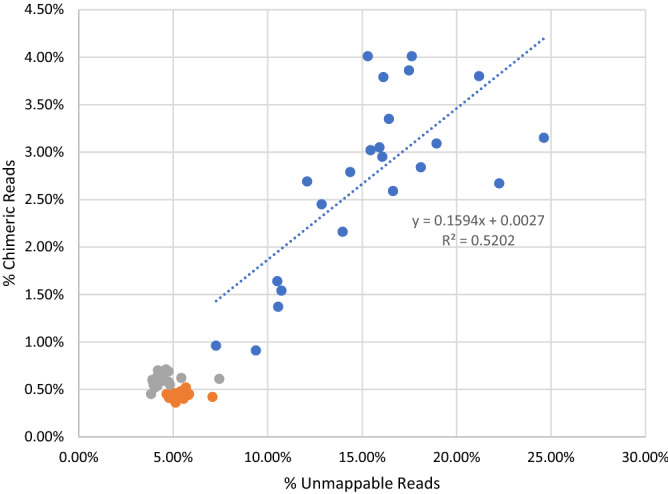
Percent chimeric reads versus percent unmappable reads for each sample. Colors correspond to different tissue types (blue = muscle, grey = abdominal fat and orange = liver). A regression line is only shown for muscle samples because the percent chimeric reads for the other tissues is negligible.

Overall, these findings suggest several muscle related genes are not represented in the current genome build, and reads for these genes are either (1) mapping to their corresponding isoforms, resulting in the reads being flagged as “too short” or (2) a paired end read is being “split” over two different isoforms as a “chimera” read (Table 2.). Muscle samples showed 4–6 times more chimeric reads than the other tissues. Further confirming this hypothesis, the percent of chimeric reads in the muscle samples was found to be moderately correlated (R^2^ = 0.5202) with the percent of unmapped reads (Fig. 2). In summary the current genome builds needs further sequencing and annotation work for muscle related genes.

**Table 2:** Mapping statistics from the 1st pass of STAR alignment for breast muscle, abdominal fat, and liver after the chimeric setting was turned on.

Tissue	Avg. uniquely mapping reads (%)	Avg. unmappable reads (too short) (%)	Avg. chimera reads (%)
Breast Muscle (n = 23)	80.11	15.39	2.73
Abdominal Fat (n = 22)	90.78	4.50	0.61
Liver (n = 23)	90.98	5.33	0.44

### Validation of variant calling pipeline

To validate the RNA-Seq pipeline, the variant calls were compared to genotyping call data produced with the same samples (Supplemental Sect. 1. Table S6). On average the panels overlapped by ~ 13,997 SNPs and genotype calls per sample had concordance of 99.46%. To verify that overall concordance was not driven by random chance or an unknown systematic error, samples were shuffled and compared to each other in a concordance matrix (Supplemental Sect. 1. Figure S4). The average concordance of randomly paired samples was 54.30%, whereas concordance of self by self was 99.50%.

### VADT analysis and results

**Figure 3:**
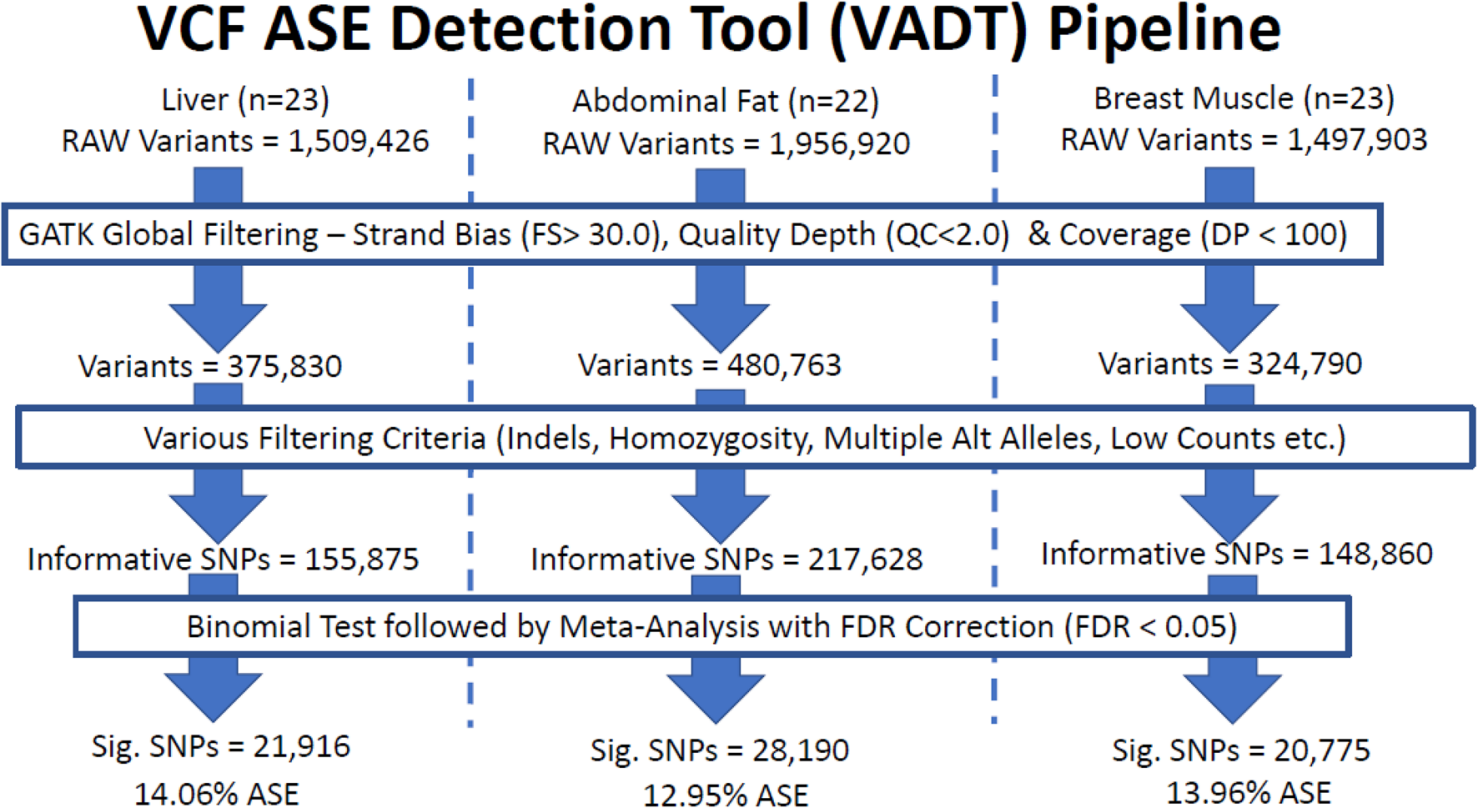
Variants detected using VCF ASE Detection Tool (VADT) pipeline for all three tissues analyzed (liver, abdominal fat and breast muscle). The pipeline consists of three major steps (blue outlined boxes): filtering using GATK (user defined filters), quality control filters and the actual statistical analysis for ASE variants.

**Figure 4:**
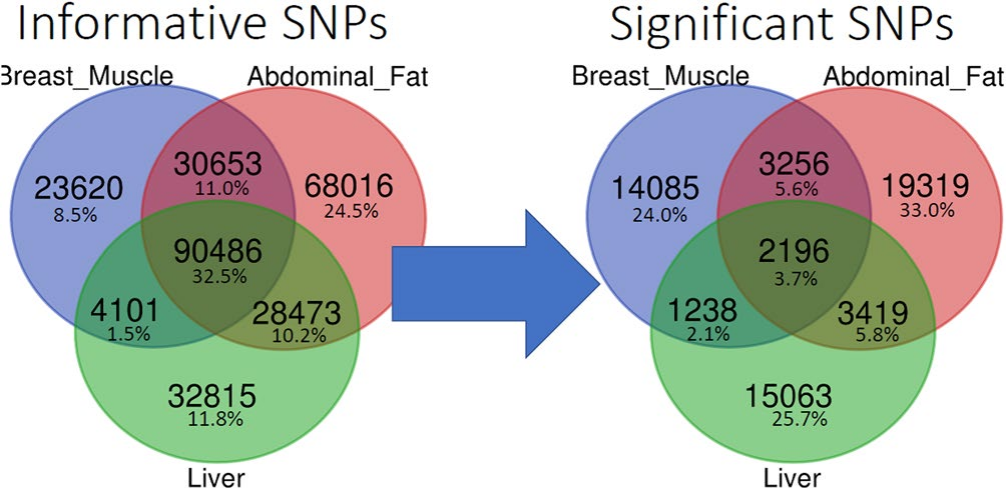
Venn diagram comparing overlapping SNPs among the three tissues. On the left are SNPs considered informative and on the right are a subset of informative SNPs that exhibit ASE. On average the informative SNPs overlap by 32.5% and ASE SNPs only overlap by 3.7%.

Between 1.5 and 2 million variants were identified in each of the three tissues. After filtering based on strand bias, quality depth and coverage, approximately 76% of variants were removed in each of the three tissues. The remaining variants were then filtered on various quality criteria as previously described (e.g. INDEL, closeness to INDELs, homozygosity, multiallelic, low counts, allele count < 1%, or no data). After filtering, the remaining informative variants were comprised of 148,860 biallelic SNPs in breast muscle, 217,628 in abdominal fat and 155,875 in liver with 32.5% of the total number of SNPs overlapping among tissues (Figs. 3 and 4). A full breakdown of summary statistics for the informative SNPs can be found in Supplemental Sect. 1. Tables S7 and S8.

We also checked for reference allele bias to assess the usefulness of masking the reference genome. This was done by comparing the reference allele ratio for informative SNPs between analyses using the masked versus unmasked reference genome. The masked reference genome reduced the reference allele ration from 51.77 to 50.17% in breast muscle, 51.93% to 50.19% in abdominal fat and 51.91% to 50.17% in liver. Reduction of the mean reference allele ratio closer to the expected ratio 50.0%, suggests that masking the reference genome helped remove reference allele bias.

The ASE analysis detected 21,916 ASE SNPs in liver, 28,190 SNPs in abdominal fat and 20,775 SNPs in breast muscle (Fig. 3). Only 2196 (3.7%) SNPs were shared among all three tissues (Fig. 4). ASE SNPs constituted approximately 13.96% of total informative SNPs for breast muscle, 12.95% for abdominal fat and 14.06% for liver.

### ASE frequency per variant

Using the multi-dimensional adjusted results, which detect individual samples exhibiting ASE for any given variant, ASE frequency (number of ASE samples/total number of samples) was calculated among all the samples and tallied on a per sample basis. It was found our overall ability to detect ASE is greatly enhanced by the larger sample size and evident by the largest ASE counts (first row) occurred only in 10% of the samples (Table 3). In other words, ASE is mostly occurring at a low frequency in the population. It is also important to note the number of ASE variants identified differ between the two statistical models implemented by VADT, seen by the lower variant count in Table 3 when compared to Fig. 3. Also, the occurrence of ASE was spread out among all samples (mean and standard deviation of number of ASE variants per sample: breast muscle 1266.30 ± 283.56, abdominal fat 1786.00 ± 277.93 and liver 1565.09 ± 215.64) with no evidence of enrichment in a few samples. It is important to note the rest of the paper focuses largely on the meta-analysis results when investigating functional significance of genes unless noted.

**Table 3:** Summary of ASE counts with corresponding percent among all the samples by ASE frequency calculated using the multi-dimensional FDR-controlling results.

	Breast muscle		Abdominal fat		Liver	
Bin	Counts	ASE percent (%)	Counts	ASE percent (%)	Counts	ASE percent (%)
0.0—0.1	13,852	84.31%	17,833	82.44%	13,850	78.58%
0.1—0.2	1534	9.34%	2349	10.86%	2155	12.23%
0.2—0.3	541	3.29%	822	3.80%	805	4.57%
0.3—0.4	310	1.89%	330	1.53%	521	2.96%
0.4—0.5	86	0.52%	165	0.76%	170	0.96%
0.5—0.6	49	0.30%	92	0.43%	71	0.40%
0.6—0.7	37	0.23%	13	0.06%	37	0.21%
0.7—0.8	11	0.07%	12	0.06%	8	0.05%
0.8—0.9	4	0.02%	10	0.05%	3	0.02%
0.9—1.0	5	0.03%	5	0.02%	5	0.03%
	Variants	16,429	Variants	21,631	Variants	17,625
	Samples	23	Samples	22	Samples	23

### Functional significance

All significant ASE SNPs were investigated for function using Ensembl’s online VEP tool. VEP returns a variety of results, such as location of the variants (exon, intron, etc.), variant’s overall consequence and gene it is predicted to have an influence on. The significant annotated variants from both models can be found in Supplemental Tables 1–6 (text files), but as prior mentioned functional analysis focuses largely on meta-analysis results only. In general, there appears to be a greater proportion of SNPs in the 3′ UTR region and downstream region of genes. A full breakdown of the SNP locations and overall consequence can be seen in Supplemental Sect. 1. Figure S5. No significant difference was identified between tissues and very few detrimental SNPs in coding regions identified with on average 0.13% (all three tissues) of variants flagged by VEP with a “high impact” consequence of “stop gain,” “stop lost” etc.

The variant effects identified from Ensembl’s VEP tool^50^ were investigated using Fisher’s Exact Test^46^. Specifically, we investigated whether there is a statistical difference between the annotations in the “Consequence” and “Impact” groups when comparing the informative variants versus the variants showing ASE based on the meta-analysis (Supplemental Sect. 1 Tables S9-14). When examining the “Impact” categories (Supplemental Tables S9-11), it quickly becomes evident there is a significant difference between the informative variants and ASE variants with some groups increasing in percentage and another group significantly decreasing. Interestingly, in the ASE variants there appears to be an enrichment in variants for “high” and “modifier” impact categories. Variants in the “high” impact category, which consisted of “splice acceptor variant”, “splice donor variant”, “start lost”, “stop gained” and “stop lost,” doubled in percentage in the ASE variants for all three tissues (avg. percentage fold change 2.17, avg. adj. p-value = 1.60 × 10^–3^). Whereas “low” impact variants actually significantly decreased in percentage in ASE variants (avg. percentage fold change 0.87, avg. adj. p-value = 1.96 x 10^–27^). This type of enrichment for functionally important variants is also evident in the “consequence” classification of variants as seen in Supplemental Sect. 1 Tables S12-14. This is evident in the significant increase in percentage of ASE variants with the following consequences of “intergenic variant” (avg. percentage fold change = 1.61, avg. adj. p-value = 7.17 x 10^–51^), “3 prime UTR variant” (avg. percentage fold change = 1.11, avg. adj. p-value = 1.23 × 10^–4^) “intron variant, non-coding transcript variant” (avg. percentage fold change = 2.11, avg. adj. p-value = 1.60 × 10^–4^) and “splice region variant, intron variant,” (avg. percentage fold change = 1.71, avg. adj. p-value = 1.66 × 10^–2^) in all three tissues. Whereas some consequences of interest that only appeared significantly in two of the three tissues with increasing percentage in ASE variants were “splice acceptor variant” (avg. percentage fold change = 5, avg adj. p-value = 1.79 × 10^–3^) in breast muscle and liver tissues and “splice donor variant” (avg. percentage fold change = 4.5, avg. adj. p-value = 1.69 × 10^–2^) and “upstream gene variant” (avg. percentage fold change = 1.07, adj. p-value = 1.61 × 10^–2^) in breast muscle and abdominal fat tissues. Whereas variants classified with the following consequences of “synonymous variant” (avg. percentage fold change = 0.86, avg adj. p-value = 3.45 x 10^–28^) and “downstream gene variant” (avg. percentage fold change = 0.90, avg. adj. p-value = 2.67 × 10^–8^) actually decreased significantly in percentage in ASE variants in all three tissues. Overall, it appears variants that may have functional influence are “enriched” in the ASE variants and variants that do not have a functional impact are decreased. The most interesting finding in this analysis is “intergenic” variants (Supplemental Sect. 1 Tables S12-14). They are showing the strongest significance and are increased in percentage in ASE variants for all three tissues, suggesting that these regions are playing an important functional role due to them being actually transcribed. As such, these intergenic variants need to be examined further.

### Pathway enrichment

The VEP results were then further parsed by Ensembl gene ID and overlapping genes between tissues examined as seen in Supplemental Sect. 1 Fig. S6, which also compares genes identified from informative SNPs. In the informative SNPs group; breast muscle captured 10,577 genes, abdominal fat captured 11,878 genes and liver captured 10,277 genes, with a high overlap of 66.9%. Whereas in the ASE SNPs group, breast muscle captured 4784 genes, abdominal fat captured 5709 genes and liver captured 4095, with an overlap of 20.1%. There was also significant enrichment of tissue specific genes.

**Figure 5:**
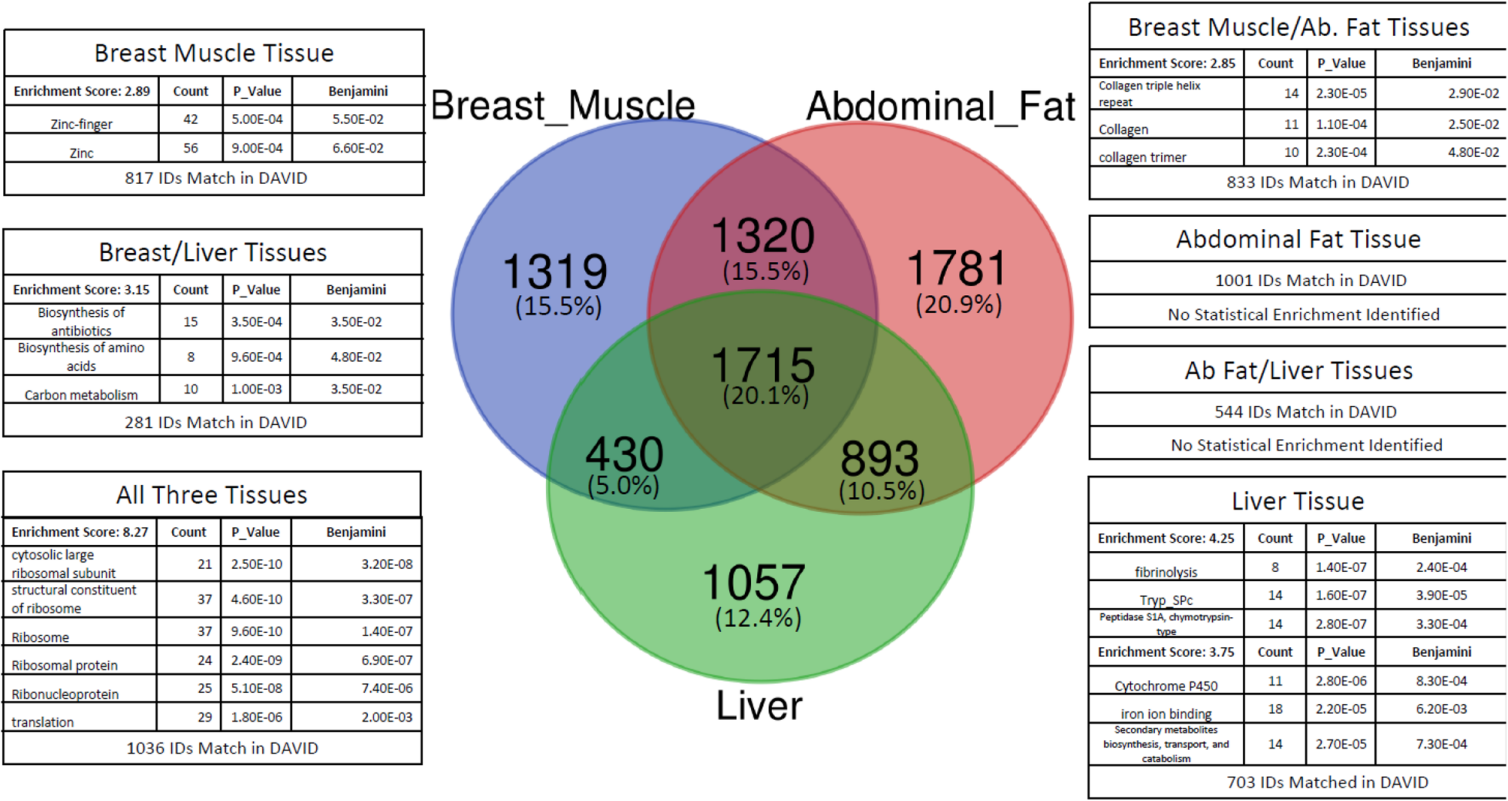
Pathway enrichment of ASE genes. The ASE variants detected in the present study were matched with their corresponding genes using Ensembl’s VEP and Ensembl gene IDs were uploaded into DAVID for biological themes enrichment analysis. The cutoff for significance was an FDR adjusted p-value ≤ 0.1 and Enrichment Score ≥ 1.3.

Genes containing ASE SNPs (i.e., ASE genes) within each tissue were submitted to DAVID for pathway enrichment. Most pathways were found to be enriched in a tissue-specific manner, although there were some exceptions (Fig. 5). The biological themes found in common for all three tissues are related to ribosomes and translation. Some groupings of genes did not show statistical significance, possibly because many Ensembl gene IDs were not identified by DAVID. The top enrichments found in DAVID for each tissue are noted in Fig. 5 and additional details are available in Supplemental Sect. 1. Tables S15-19.

### Identification of robust ASE genes found in all three tissues

**Figure 6:**
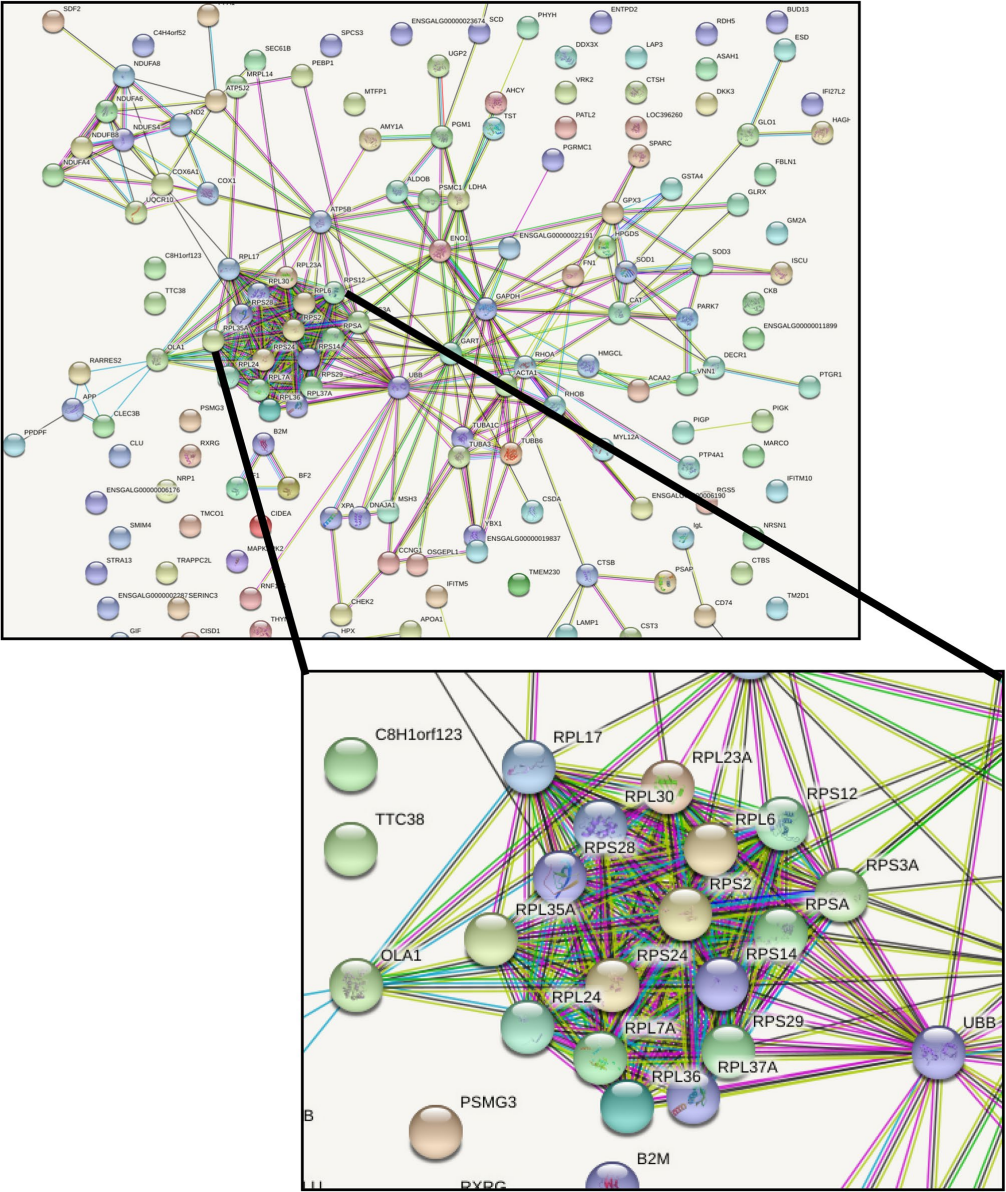
Network analysis of genes enriched for ASE across all three tissues. Genes that showed a 3-tissue average ASE score of 50% (or higher) after normalization were submitted to STRING (n = 208) to examine protein interactions. There were a total 176 nodes, 374 edges with an average node degree 4.25 with a PPI enrichment p-value of < 1.0e−16 (significant interactions identified). The largest network identified in the analysis was ribosomal genes (zoomed in image).

For additional functional analyses, we focused on ASE genes that were common to all three tissues and showed the strongest ASE evidence. In all three tissues, ASE genes were investigated by normalizing the number of ASE SNPs found in a gene to its total number of informative SNPs by utilizing the following equation (ASE SNPs/informative SNPs) × 100 = normalized gene ASE score. The normalized gene ASE score among the three tissues was then averaged to create a global (across tissue) gene ASE score. Out of the 1715 ASE genes found in common among all tissues, a total of 344 genes had a global ASE score greater than or equal to 50%, and of these 344 genes, 208 genes had assigned gene symbols. These 208 genes were uploaded to STRING^56^ to examine protein interactions (Fig. 6). Ribosomal genes formed the largest and most interconnected network of interactions among all the genes. The top three KEGG pathways^57^ identified by STRING were ribosomes (count = 18, FDR adjusted p-value = 8.62e−15), metabolic pathways (count = 32 FDR adjusted p-value = 3.33e−07) and oxidative phosphorylation (count = 11, FDR adjusted p-value = 8.93e−07). When a more stringent cutoff of ≥ 80% is applied, 78 genes were identified, and this full list can be found in Supplemental Sect. 1. Table S20. Out of the entire list, 23 genes had a global ASE scores of 100%, of which 10 genes have identifiable gene symbols that could be referenced for biological function in GeneCards^58^. These top 10 genes with corresponding function can be seen in Table 4. In the list, 3 out of 10 genes are ribosomal protein coding genes (*RPS29*, *RPL35A* and *MRPL43*) and 3 out of 10 genes are directly involved in metabolism (*UBL5*, *RARES2* and *MT-ND2*).

**Table 4:** Genes with the strongest ASE evidence identified across three tissues using VADT and Ensemble’s VEP tool.

Ensembl gene ID	Gene symbol	Gene name	Biological function
ENSGALG00000021139	IGLL1	Immunoglobulin Lambda Like Polypeptide 1	Cell surface receptor involved in signal transduction for proliferation and differentiation of ProB cell to preB cell
ENSGALG00000029837	OST4	Oligosaccharyltransferase Complex Subunit 4, Non-Catalytic	Forms part of complex that transfers oligosaccharides to polypeptide chains
ENSGALG00000035138	UBL5	Ubiquitin Like 5	Codes a protein that functions like ubiquitin, but instead bind to a protein and interfere with its function and appears to show association with metabolism
ENSGALG00000007611	RPL35A	Ribosomal Protein L35A	Codes a protein that forms part of the 60S subunit of ribosomes
ENSGALG00000008066	UQCR10	Ubiquinol-Cytochrome C Reductase, Complex III Subunit X	Codes for a subunit of protein that forms part of the inner mitochondrial membrane
ENSGALG00000034352	MRPL43	Mitochondrial Ribosomal Protein L43	Codes for a protein that forms part of subunit of mitochondrial ribosomes
ENSGALG00000012229	RPS29	Ribosomal Protein S29	Codes for protein that forms part of the 40S subunit of ribosomes
ENSGALG00000042642	RARRES2	Retinoic Acid Receptor Responder 2	Receptor which helps transmits signaling for the regulation of various biological functions like adipogenesis, metabolism and inflammation
ENSGALG00000021365	DCTN3	Dynactin Subunit 3	Forms a sub-unit that is part of the dynactin protein complex. Dynactin are involved in a variety of functions associated with microtubules
ENSGALG00000043768	MT-ND2	Mitochondrially Encoded NADH: Ubiquinone Oxidoreductase Core Subunit 2	Forms an important subunit in mitochondria that is involved NADH dehydrogenase, which is extremely important in metabolism

Biological function is based on GeneCards^58^.

### Examination of ribosomal variants show variable penetrance and strength of ASE

To further examine the ribosomal protein coding genes, the ASE variants for the three ribosomal genes identified in Table 4. (*RPS29*, *RPL35* and *MRPL43*) were inspected in greater detail. This included investigating the meta-data of samples (high and low feed efficiency status of birds) and verification of ASE statistical significance in individual samples based on the multi-dimensional-FDR corrected analysis for these variants (Table 5). Utilizing the meta-data, the feed efficiency status (HFE vs LFE) and ASE were investigated for statistical enrichment first using Student’s T-test comparing the groups in Table 5, which showed no statistical difference. The study was then expanded to using Fisher’s Exact Test to examine the meta-data based on feed efficiency status for all variants not just ribosomal genes (Supplemental Sect. 1 Tables S21-23), but no variant reached statistical significance possibly due to overall data dimensionality, and complex nature of the trait, requiring a larger sample size to capture the complexity. As seen in Table 5. it becomes evident that each variant showed a different ASE frequency among the samples, although an individual variant’s overall behavior was consistent between tissues. For example, variant chr5:57721190, found in the *RPS29* ribosomal gene, showed consistently high frequency of ASE among biallelic samples in all three tissues, which had a consistently strong signal for ASE (statistically significant for ASE). In contrast, variant chr6:23089484 shows low frequency of ASE on a per-sample basis in all three tissues, as evidenced by liver tissue having no statistically significant samples and the other two tissues having only one significant sample. It is important to note this variant was properly flagged as showing ASE in all three tissues by the meta-analysis, but by utilizing results from the multi-dimensional analysis further interpretation of the data can occur. Also, seen in Table 5 is consistency in the directionality of ASE, which allele is higher expressed, is consistent between samples for a specific variant. When this behavior was investigated globally, utilizing the multi-dimensional FDR-controlling results, it was found variants generally showed high concordance for a specific allele (concordance for each tissue: breast muscle = 95.17%, abdominal fat = 94.84% and liver = 94.07%).

**Table 5:** Examination of ribosomal genes and their corresponding variants identified as showing ASE from the meta-analysis.

Tissue (n)	Gene symbol	Location	Variant	Biallelic^a^	ASE^b^	ASE Allele	HFE (n)	LFE (n)
Breast muscle (HFE = 10, LFE = 13)	RPS29	chr5:57721190	rs733556517	15	15	Alt	6	9
	RPS29	chr5:57721198	–	2	2	Ref	1	1
	RPL35A	chr9:15172542	rs3137208	6	6	Alt	4	2
	MRPL43	chr6:23089484	–	11	1	Ref	1	0
Abdominal fat (HFE = 10, LFE = 12)	RPS29	chr5:57721190	rs733556517	16	16	Alt	8	8
	RPS29	chr5:57721198	–	3	3	Ref	3	0
	RPL35A	chr9:15172542	rs3137208	3	3	Alt	2	1
	MRPL43	chr6:23089484	–	11	1	Ref	0	1
Liver (HFE = 11, LFE = 12)	RPS29	chr5:57721190	rs733556517	14	13	Alt	7	6
	RPS29	chr5:57721198	–	4	3	Ref	2	1
	RPL35A	chr9:15172542	rs3137208	6	6	Alt	2	4
	MRPL43	chr6:23089484	–	12	0	No sig. indiv. samples		

Included is the breakdown of the meta-data for those corresponding samples (HFE = “High-Feed Efficiency” and LFE = “Low-Feed Efficiency”) and the multi-dimensional FDR corrected sample results that showed statistically significant ASE.

^a^Number of samples expressing both alternative and reference alleles of the variant.

^b^Number of samples showing ASE.

## Discussion

ASE tissue specificity in chickens was previously reported by Zhuo et al*.*^13^ and in other organisms^59–64^. ASE was recently reported to occur frequently in bovine muscle and in genomic regions associated with economically important traits^65^. The present study is the first to show ASE in non-tissue specific genes in chickens, specifically genes involved in ribosomes and translational machinery. This mechanism was previously reported in hybrid catfish in 2016, which are bred for production traits in aquaculture^66^.

Modern breeding for broiler chickens value fast growth, high yield, and high feed efficiency^67^. This selective pressure has mostly caused upregulation of key genes and pathways that are involved in metabolism and protein production to favor greater growth and efficiency. It is possible that ASE SNPs, especially those identified in ribosomal genes are associated with beneficial improvements in translation, however, without eQTL data, this is just speculative. The presence of ASE in ribosomal genes in all three tissues, most likely, implies the *cis-*regulatory mechanism for these variants is not tissue specific. Therefore, when selecting for key ASE variants for future breeding purposes various factors must be considered like the prevalence and strength of the ASE variant along with its tissue specificity. Though, ASE may also result from genomic imprinting, where expression of one of the two alleles is silenced depending on the parental origin, recent studies have shown no evidence of imprinting occurring in chickens^13^.

In conclusion, this study presents a new analysis pipeline that simplifies and standardizes ASE detection. We have also showed the impact of ASE SNPs in chickens especially their potential role in translational machinery. Additional research is necessary to fully understand the biological mechanisms underlying ASE variants and their importance for economically important traits in poultry production.

VADT is a streamlined ASE detection pipeline that is robust, accurate and straight-forward to run. The pipeline follows GATK’s Best Practices to produce an initial VCF, which can be fed directly into VADT for ASE detection. The output from VADT can easily be uploaded to VEP parser for other downstream analyses. Understanding the biological effect of ASE is key to gaining a better understanding of *cis*-regulatory elements in the genome.

Our analysis pipeline for SNP calling was found to be highly accurate as seen by the strong correlation with our DNA genotyping calls. However, a large number of SNPs were excluded due low overlap with the 600K chicken genotyping panel. This low overlap between the RNA-seq variant calls and genotyping panel is most likely due to limitations of commercial genotyping panels in general. Genotyping panels have a limited number of variants spread across the entire genome that must be applicable to a broad genetic background. However, the high concordance of overlapping SNPs validates the use of VADT for ASE SNP detection. The biggest problem we encountered with our entire analysis pipeline was mapping issues with muscle tissue samples. This limitation was most likely due to current genome build and its annotation of muscle related genes.

VADT is a self-contained python-based program that requires minimal effort from a user to run, the only requirement is a high-performance computing (HPC) environment due to the size of and complexity of VCF files. VADT is the first ASE detection program that does not require any modifications of user input files and has FDR correction built in, allowing users to focus more on the biological significance of their results. VADT was tested and validated on a different dataset (data not included) and proven to be robust and accurate. Also, VADT allows a user to analyze multiple tissues in a high throughput manner and with less susceptibility to user error. The ability to use VADT with two different statistical models allows users to thoroughly investigate ASE behavior on both a per variant and per sample basis, allowing for a greater understanding of ASE and its biological impact and regulation.

## Supplementary Information

Supplementary Information 1.

Supplementary Information 2.

Supplementary Tables.
