# Supplementary material for: Investigation of allele specific expression in various tissues of broiler chickens using the detection tool VADT
Source: Sci Rep. 2021 Feb 17;11:3968. doi: 10.1038/s41598-021-83459-8 (PMC7889858; doi:10.1038/s41598-021-83459-8)
Supplement: Supplementary file 1 — Supplementary Information 1. [file 41598_2021_83459_MOESM1_ESM.docx]

**Investigation of Allele Specific Expression in Various Tissues of Broiler Chickens Using the Detection Tool VADT**

**M. Joseph Tomlinson IV ^§1, 5^, Shawn W. Polson^2,3,5^, Jing Qiu^4,5^, Juniper A. Lake^1,5^, William Lee^,6^, Behnam Abasht*^1,5^**

**Affiliations**

1. Department of Animal and Food Sciences, University of Delaware

2. Department of Computer and Information Sciences, University of Delaware

3. Department of Biological Sciences, University of Delaware

4. Department of Applied Economics and Statistics, University of Delaware

5. Center for Bioinformatics and Computational Biology, University of Delaware

6. Maple Leaf Farms, Inc. Leesburg, IN 46538, USA

**^§^** First Author

* Corresponding author: abasht@udel.edu

1. Department of Animal and Food Sciences, University of Delaware, 531 South College Ave, Newark, DE 19716, USA

**Supplemental Section 1**

**Samples Utilized for ASE Analysis by Tissue**

Liver (n = 23)

Abdominal Fat (n = 22)^*^

Breast Muscle (n = 23)^**^


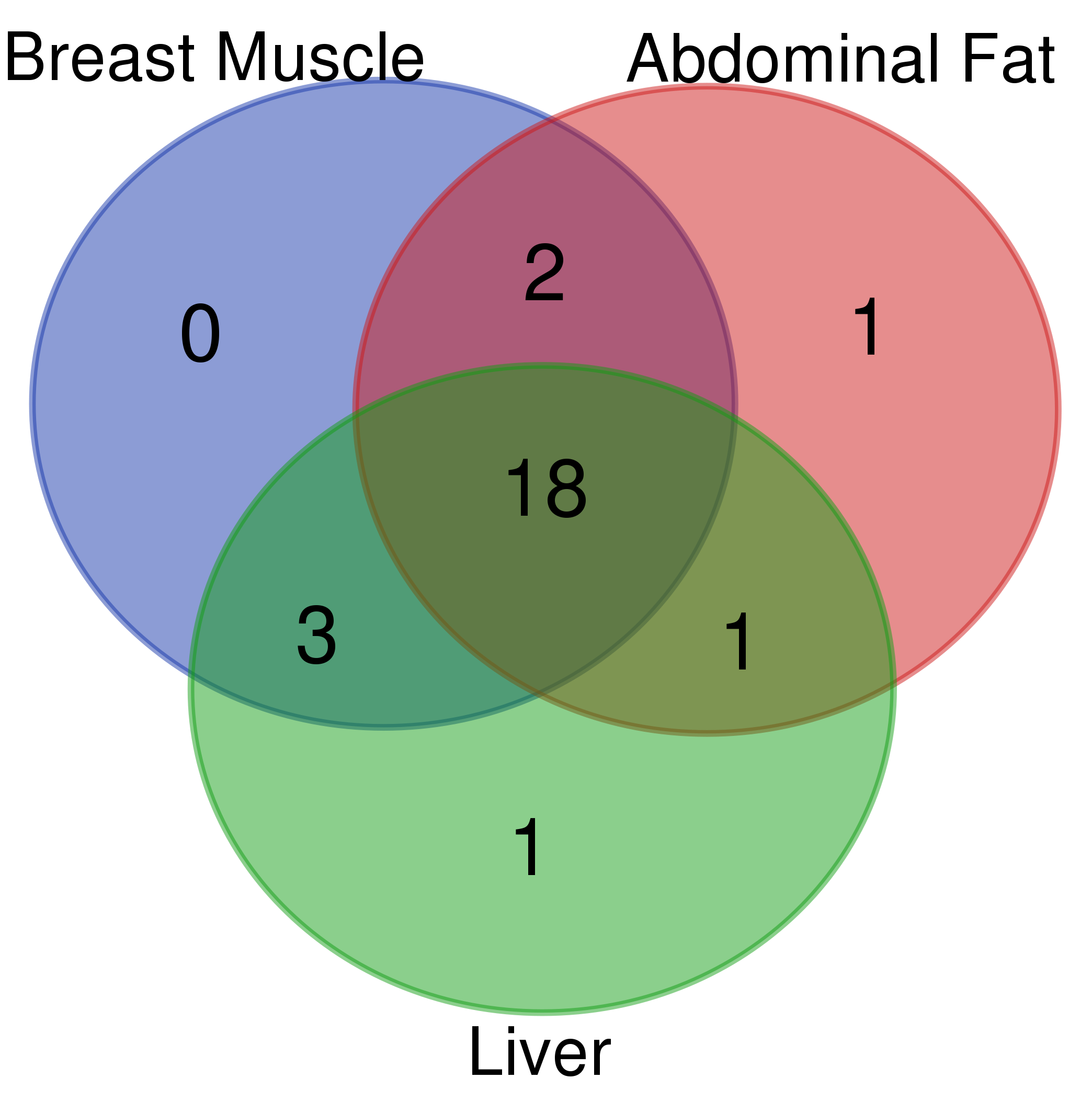


**Supplemental Figure S1.** Samples used in this ASE study with corresponding overlap in various tissues. *Samples utilized in study by Zhuo et al (2015) [1]. **Samples utilized in study by Zhou et al (2015) [2].

**Supplemental Figure S2.** RNA-seq variant calling pipeline. The pipeline consists of three parts: part 1. the initial calling of variants using an unmasked genome sequence, part 2. variants were used to create a global g.vcf file that was used to create a masked genome sequence and part 3. repeating the variant calling pipeline but aligning with the masked genome sequence to help remove reference allele bias [3-10].

**Supplemental Table S1.** Summary statistics of STAR alignment (1^st^ Pass) for all the samples separated by project that were used to create the initial VCF used for masking. The additional 32 samples utilized in the initial variant calling had a diversity of input lengths (*) and represent the average between the samples. These additional samples were not later utilized in the ASE analysis.

| **Feed Efficiency Project Samples** | | | | |
| --- | --- | --- | --- | --- |
|  | Avg. Input Reads | Input Length | Avg. Uniquely Mapped Reads | % Uniquely Mapped Reads |
| Breast Muscle (n=23) | 34212079.35 | 150 | 27477522.91 | 80.11% |
| Abdominal Fat (n=22) | 32096465.36 | 150 | 29137588.18 | 90.78% |
| Liver (n=23) | 33306427.57 | 150 | 30305271.57 | 90.98% |
| All Samples (n=68) | 33221292.6 | 150 | 28971047.25 | 87.24% |
|  | | | | |
| **Additional Samples Utilized in Variant Calling** | | | | |
|  | Avg. Input Reads | Input Length | Avg. Uniquely Mapped Reads | % Uniquely Mapped Reads |
| Breast Muscle (n=24) | 51070107.71 | 174.42* | 42340701.46 | 82.94% |
| Liver (n=8) | 16858849.63 | 202 | 13961441.13 | 83.85% |
| All Samples (n=32) | 42517293.19 | 181.31* | 35245886.38 | 83.17% |

Abdominal Fat

Breast Muscle

Liver

**Supplemental Figure S3.** Examination of RNA-Seq gene coverage for all samples using RSeQC’s (version 2.6.4) geneBody_coverage.py [11]. No systematic bias was identified in overall coverage when comparing breast muscle to liver and abdominal fat.

**Supplemental Table S2.** Overall summary statistics from the unmappable reads for R1 and R2 (forward and reverse primers) for all three tissues in sample 47337. The “Average Trimmed Length” is the average sequence length after trimming by FastqBLAST and the “Average Hit Seq. Length” is the length BLAST returned for the match. The “Average Hit Seq. Length” could be larger than the prior trimmed length due to sequences not being found during the BLAST.

| **FastqBLAST Results** | | | | | |
| --- | --- | --- | --- | --- | --- |
|  | **Sequences in File** | **BLASTed** | **Avg. Trimmed Length** | **Blast Hits** | **Avg. Hit Seq. Length** |
| Muscle R1 | 5,735,211 | 1000 | 70.54 | **736** | 70.19 |
| Muscle R2 | 5,735,211 | 1000 | 57.58 | **608** | 69.19 |
| Abdominal Fat R1 | 1,882,124 | 1000 | 70.72 | 544 | 68.64 |
| Abdominal Fat R2 | 1,882,124 | 1000 | 60.99 | 415 | 66.14 |
| Liver R1 | 1,821,565 | 1000 | 70.48 | 734 | 70.84 |
| Liver R2 | 1,821,565 | 1000 | 43.75 | 330 | 66.76 |

**Supplemental Table S3.** Top 5 FastqBLAST count results for R1 and R2 of unmappable reads for the three tissues (breast muscle, abdominal and liver) for sample 47337

| **Top Muscle Genes from Unmappable Reads (R1)** | | |
| --- | --- | --- |
| **Accession ID** | **Description** | **Counts** |
| NM_205119.1 | Gallus gallus enolase 3 (beta, muscle) (ENO3), mRNA >gi | 51 |
| NM_001198744.1 | Gallus gallus myosin light chain, phosphorylatable, fast skeletal muscle (MYLPF), mRNA >gi | 46 |
| NM_205519.1 | Gallus gallus ATPase sarcoplasmic/endoplasmic reticulum Ca2+ transporting 1 (ATP2A1), mRNA >gi | 46 |
| NM_205507.1 | Gallus gallus creatine kinase, M-type (CKM), mRNA >gi | 41 |
| KY039437.1 | Gallus gallus isolate ACAD15101_Palawan_Philippines mitochondrion, complete genome | 33 |
| **Total** | | **217** |
|  |  |  |
| **Top Muscle Genes from Unmappable Reads (R2)** | | |
| **Accession ID** | **Description** | **Counts** |
| NM_205519.1 | Gallus gallus ATPase sarcoplasmic/endoplasmic reticulum Ca2+ transporting 1 (ATP2A1), mRNA >gi | 44 |
| NM_205119.1 | Gallus gallus enolase 3 (beta, muscle) (ENO3), mRNA >gi | 39 |
| NM_205507.1 | Gallus gallus creatine kinase, M-type (CKM), mRNA >gi | 39 |
| NM_001198744.1 | Gallus gallus myosin light chain, phosphorylatable, fast skeletal muscle (MYLPF), mRNA >gi | 37 |
| XM_025151021.1 | PREDICTED: Gallus gallus creatine kinase M-type-like (LOC107051134), partial mRNA | 23 |
| **Total** | | 182 |
|  |  |  |
| **Top Abdominal Fat Genes from Unmappable Reads (R1)** | | |
| **Accession ID** | **Description** | **Counts** |
| XR_003078040.1 | PREDICTED: Gallus gallus 28S ribosomal RNA (LOC112533599), rRNA | 31 |
| XR_003078044.1 | PREDICTED: Gallus gallus 18S ribosomal RNA (LOC112533603), rRNA | 23 |
| BX931917.1 | Gallus gallus finished cDNA, clone ChEST790c21 | 12 |
| XM_025155812.1 | PREDICTED: Gallus gallus avian tenascin X (TNX), transcript variant X18, mRNA | 9 |
| XR_003078043.1 | PREDICTED: Gallus gallus 18S ribosomal RNA (LOC112533602), rRNA | 8 |
| **Total** | | **83** |
|  |  |  |
| **Top Abdominal Fat Genes from Unmappable Reads (R2)** | | |
| **Accession ID** | **Description** | **Counts** |
| XR_003078040.1 | PREDICTED: Gallus gallus 28S ribosomal RNA (LOC112533599), rRNA | 38 |
| XR_003078044.1 | PREDICTED: Gallus gallus 18S ribosomal RNA (LOC112533603), rRNA | 16 |
| BX931917.1 | Gallus gallus finished cDNA, clone ChEST790c21 | 13 |
| XM_025155812.1 | PREDICTED: Gallus gallus avian tenascin X (TNX), transcript variant X18, mRNA | 7 |
| KY039437.1 | Gallus gallus isolate ACAD15101_Palawan_Philippines mitochondrion, complete genome | 7 |
| **Total** | | 81 |

|  |  |  |
| --- | --- | --- |
| **Top Liver Genes from Unmappable Reads (R1)** | | |
| **Accession ID** | **Description** | **Counts** |
| XR_003078040.1 | PREDICTED: Gallus gallus 28S ribosomal RNA (LOC112533599), rRNA | 74 |
| XM_025149533.1 | PREDICTED: Gallus gallus albumin (ALB), transcript variant X5, mRNA | 45 |
| KY039437.1 | Gallus gallus isolate ACAD15101_Palawan_Philippines mitochondrion, complete genome | 35 |
| XM_025144502.1 | PREDICTED: Gallus gallus cytochrome P450 2G1-like (LOC112530469), mRNA | 34 |
| U16848.1 | GGU16848 Gallus gallus complement C3 precursor mRNA, complete cds | 30 |
| Total |  | 218 |
|  |  |  |
| **Top Liver Genes from Unmappable Reads (R2)** | | |
| **Accession ID** | **Description** | **Counts** |
| XR_003078040.1 | PREDICTED: Gallus gallus 28S ribosomal RNA (LOC112533599), rRNA | 60 |
| XM_025144502.1 | PREDICTED: Gallus gallus cytochrome P450 2G1-like (LOC112530469), mRNA | 30 |
| XR_003078044.1 | PREDICTED: Gallus gallus 18S ribosomal RNA (LOC112533603), rRNA | 24 |
| U16848.1 | GGU16848 Gallus gallus complement C3 precursor mRNA, complete cds | 20 |
| XM_025149533.1 | PREDICTED: Gallus gallus albumin (ALB), transcript variant X5, mRNA | 8 |
| Total |  | 142 |

**Supplemental Table S4.** Top 15 FastqBLAST count results for muscle’s R1 and R2 for sample 47337 with corresponding results if gene was found in GTF. GTF files for both Gallus_gallus_5.0 (GTF 86) and Gallus_gallus_6.0. (GTF 98) were compared and found to show similar results [8, 12]. It should be recognized exact term matches were required to count as a hit, due to this stringency numerous genes had “No Hit”, but potentially could have isoform matches. A gene designated as “Hit” was found in the GTFs and was further explored for mapping issues below the table. Numbers in table correspond to possible mapping issue. A gene with a “?” was designated where no clear gene name was identifiable in the description from BLAST.

**Supplemental Table S4. (Description Above)**

| Accession ID | Description | Counts R1 | Counts R2 | Term Searched | GTF Bld5.0.0 | GTF Bld6.0.0 | Possible Mapping Issue |
| --- | --- | --- | --- | --- | --- | --- | --- |
| NM_205119.1 | Gallus gallus enolase 3 (beta, muscle) (ENO3), mRNA >gi | 51 | 39 | ENO3 | No Hit | No Hit |  |
| NM_205519.1 | Gallus gallus ATPase sarcoplasmic/endoplasmic reticulum Ca2+ transporting 1 (ATP2A1), mRNA >gi | 46 | 44 | ATP2A1 | No Hit | No Hit |  |
| NM_001198744.1 | Gallus gallus myosin light chain, phosphorylatable, fast skeletal muscle (MYLPF), mRNA >gi | 46 | 37 | MYLPF | No Hit | No Hit |  |
| NM_205507.1 | Gallus gallus creatine kinase, M-type (CKM), mRNA >gi | 41 | 39 | CKM (1) | Hit | Hit | 1 |
| KY039437.1 | Gallus gallus isolate ACAD15101_Palawan_Philippines mitochondrion, complete genome | 33 | 14 | ? |  |  |  |
| XM_015284056.2 | PREDICTED: Gallus gallus actin, alpha 1, skeletal muscle (ACTA1), transcript variant X1, mRNA | 27 | 19 | ACTA1 | One Hit | One Hit | 2 |
| XM_025151021.1 | PREDICTED: Gallus gallus creatine kinase M-type-like (LOC107051134), partial mRNA | 25 | 23 | See Prior CKM Entry | | | |
| NM_001044659.1 | Gallus gallus myosin binding protein C, fast type (MYBPC2), mRNA >gi | 23 | 20 | MYBPC2 | No Hit | No Hit |  |
| XM_025152554.1 | PREDICTED: Gallus gallus nebulin-like 1 (NEBL1), transcript variant X29, mRNA | 14 | 10 | NEBL1 | No Hit | No Hit |  |
| AY466166.1 | Gallus gallus clone KNC-NDS6 unknown mRNA | 13 | 16 | ? |  |  |  |
| XR_003078040.1 | PREDICTED: Gallus gallus 28S ribosomal RNA (LOC112533599), rRNA | 11 | 10 | 28S | No Hit | No Hit |  |
| XM_025148126.1 | PREDICTED: Gallus gallus glycogen phosphorylase, muscle form-like (LOC107049660), mRNA | 10 | 11 | PYGM | No Hit | No Hit |  |
| NM_204305.1 | Gallus gallus glyceraldehyde-3-phosphate dehydrogenase (GAPDH), mRNA >gi | 9 | 14 | GAPDH | Hit | Hit | 3 |
| XM_015286580.2 | PREDICTED: Gallus gallus lactate dehydrogenase A (LDHA), transcript variant X1, mRNA | 9 | 8 | LDHA | Hit | Hit | 4 |
| NM_001013397.2 | Gallus gallus myosin, heavy chain 1E, skeletal muscle (MYH1E), mRNA | 8 | 10 | MYH1E | Hit | Hit | 5 |

**Possible Mapping Issue Further Explored**

1. *CKM* has two isoforms *CKMT2* (ENSGALG00000015602 chrZ) and *CKMT1B* (ENSGALG00000008352 chr10), which are found on different chromosomes.
2. *ACTA1*(ENSGALG00000011086 chr3) shows sequence similarity to *ACTA2* (ENSGALG00000006343 chr6), with a BLASTN percent identity of 83.95% (compared mRNA sequences).
3. *GAPDH* (ENSGALG00000014442 chr1) has many pseudogenes that may cause mapping related issues [13].
4. *LDHA* (ENSGALG00000006300 chr5) shows sequence similarity to *LDHB* (ENSGALG00000035836 chr1), with a BLASTN percent identity of 73.75% (compared mRNA sequences).
5. *MYH1E* (ENSGALG00000029606 chr18), the myosin heavy chain (MHC) has numerous isoforms [14]. If the term “MYH” is searched in the GTF files 9 isoforms are found: *MYH1E* (ENSGALG00000029606 chr18), *MYH11* (ENSGALG00000006520 chr14), *MYH1D* (ENSGALG00000027323 chr18), *MYH1F* (ENSGALG00000042257 chr18), *MYH1A* (ENSGALG00000037864 chr18), *MYH1B* (ENSGALG00000039977 chr18), *MYH1C* (ENSGALG00000032404 chr18), *MYH10* (ENSGALG00000001183 chr18), *MYH15* (ENSGALG00000015358 chr1).

**Supplemental Table S5.** Comparison of mapping rates between genome builds after STAR alignment (1^st^ Pass) [5] for the various tissues for sample 47337. Overall analysis using latest genome build followed same procedure prior implemented but with latest references when aligning [12, 15].

| **Sample 47337** | | |
| --- | --- | --- |
| **Tissue** | **Gallus gallus 5.0** | **Gallus gallus 6.0** |
| Liver | 91.42 | 94.1 |
| Abdominal Fat | 91.25 | 93.32 |
| Breast Muscle | 79.43 | 80.92 |

**Supplemental Table S6.** Comparison of SNP calls between 600K Genotyping panel and RNA-seq SNPs. The initial total SNPs counts for both the panel and RNA-seq are based on SNP calls after filtering and represent all high-quality SNPs that can be compared between the datasets.

|  | **Muscle** | **Liver** | **Abdominal Fat** |
| --- | --- | --- | --- |
| Number of Samples | 23 | 23 | 22 |
| Total Number of SNPs in Genotyping Panel | 572,645 | 572,645 | 572,645 |
| Total Number of SNPs in RNA-Seq Dataset | 193,245 | 199,330 | 268,744 |
|  |  |  |  |
| Total Overlapping SNPs Between Genotyping Panel and RNA-Seq | 12,769 | 12,764 | 16,459 |
|  |  |  |  |
| **Genotype Matching Stats**  **(For the overlapping SNPs Across All Samples)** | | | |
| Total Matches | 168,885 | 187,463 | 254,846 |
| Total Non-matches | 997 | 962 | 1,323 |
| Concordance (%) | 99.41 | 99.49 | 99.48 |
|  |  |  |  |
| **Non-Matching Stats** | | | |
| Homozygous Ref Allele | 299 | 362 | 401 |
| Homozygous Alt Allele | 131 | 104 | 152 |
| Discordant Genotype (new allele) | 567 | 496 | 770 |

**A.** Concordance Matrix Comparison of Breast Muscle Samples


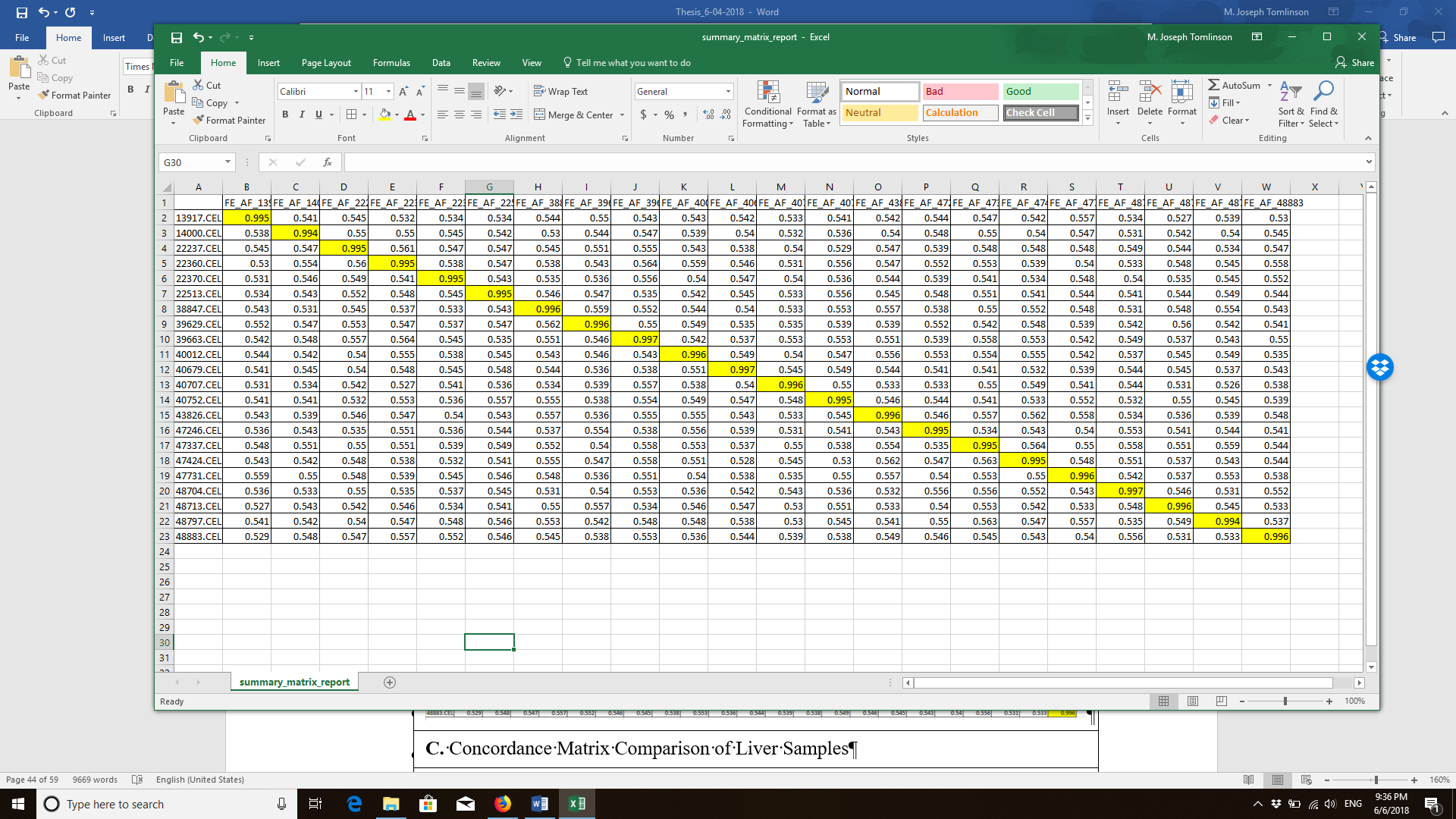


**B.** Concordance Matrix Comparison of Abdominal Fat Samples


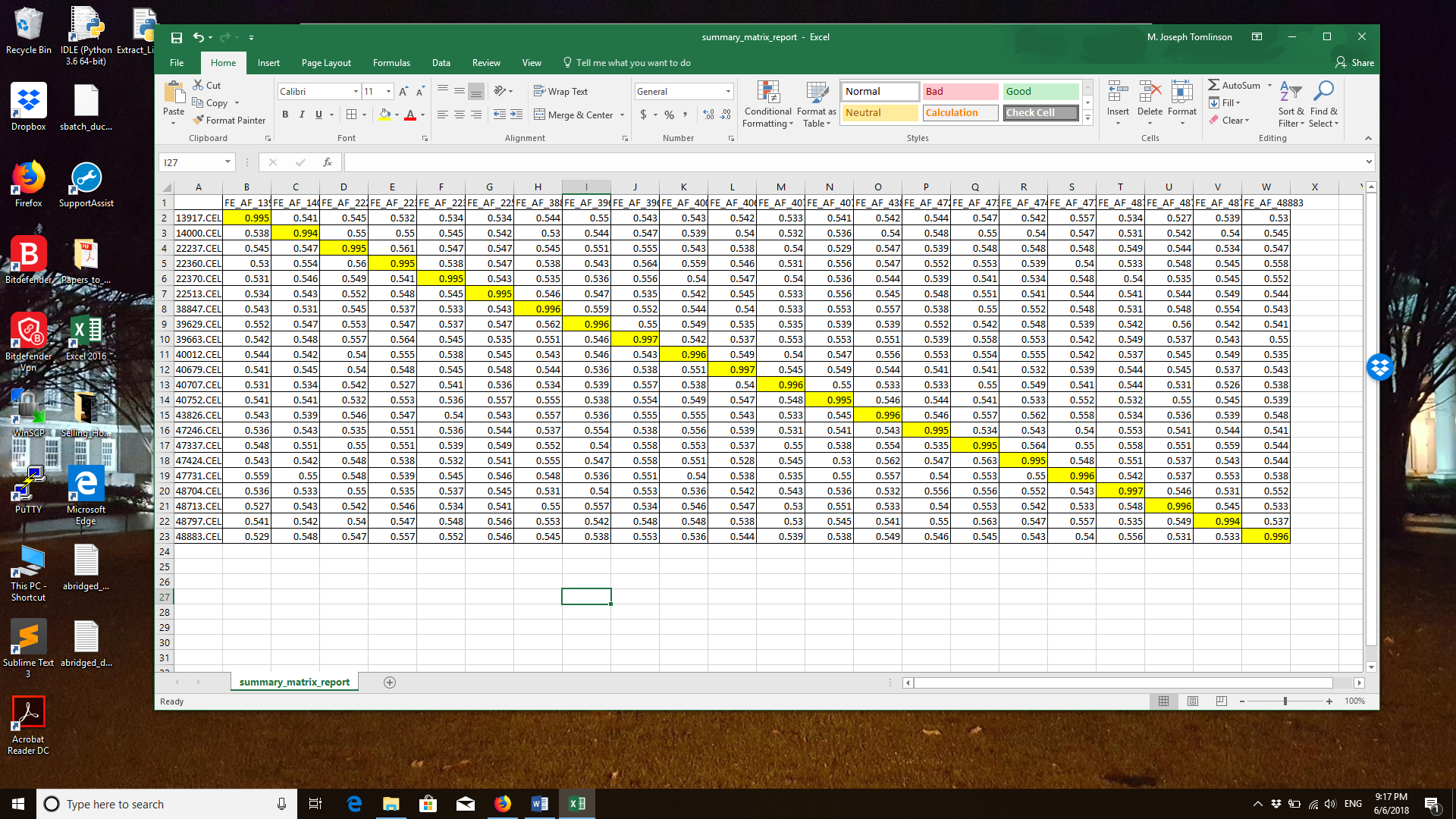


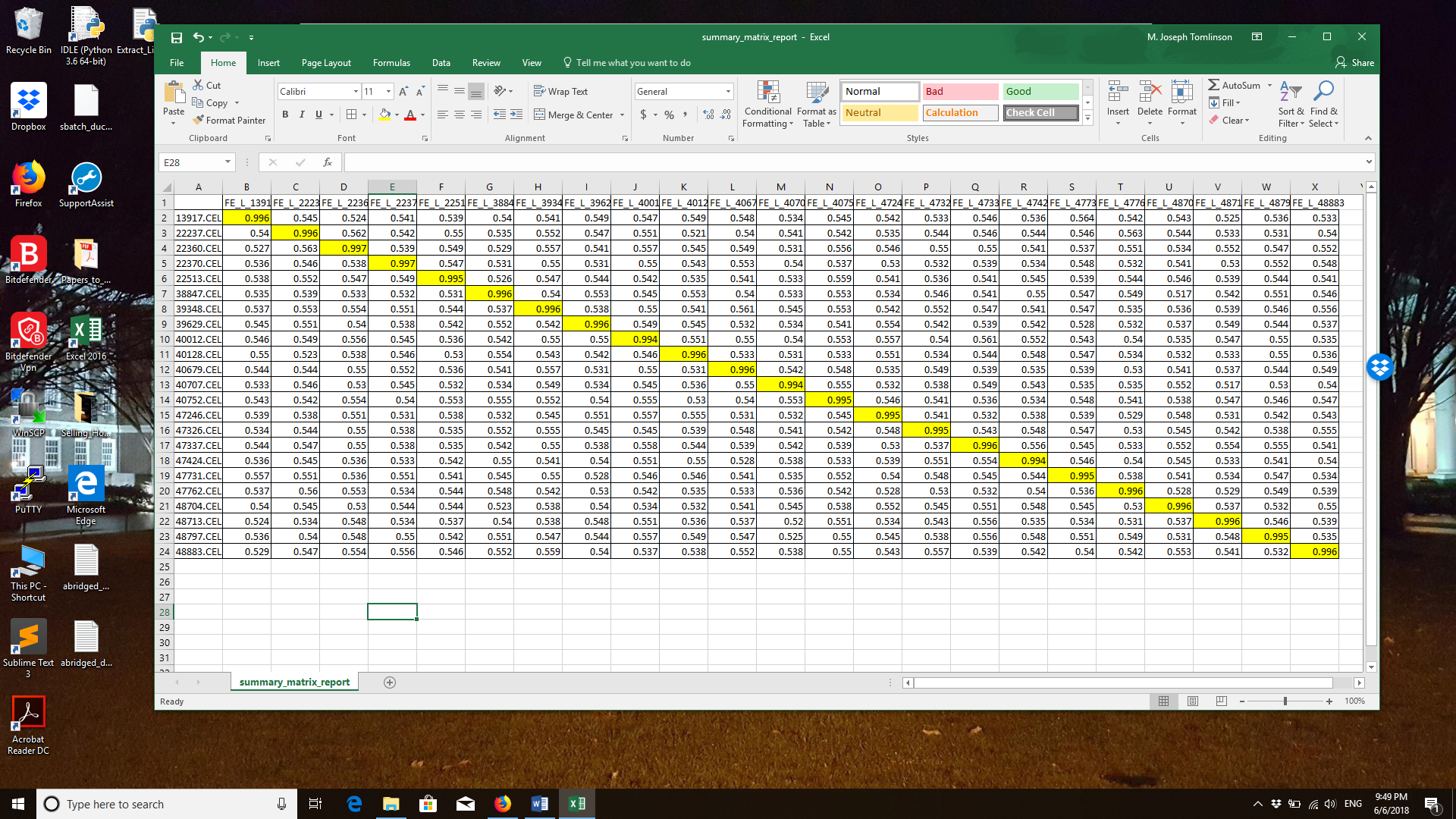


**C.** Concordance Matrix Comparison of Liver Samples

**Supplemental Figure S4**. Matrix comparisons of genotype calls for 600K versus RNA-seq for samples separated by tissue type. On the y-axis is the 600K genotyping dataset and on the x-axis the RNA-Seq dataset. Diagonal lines (highlighted in yellow) represent self to self. Average scores tissue type: muscle-self = 0.995, muscle-rest = 0.543, abdominal fat-self = 0.996, abdominal fat-rest = 0.544, liver-self = 0.995, liver-rest = 0.542.

**Breakdown of InfORMATIVE SNP Counts**

Informative SNP counts were analyzed for overall summary statistics and broken down by tissue and can be seen in the following two tables, Supplemental Table S7 consists of summary stats about the read counts whereas Supplemental Table S8 breaks down the binning of read counts. It was found the majority of SNPs have read counts between 20-200 counts.

**Supplemental Table S7.** Summary statistics of informative SNPs read counts per tissue.

|  | **Breast Muscle** | **Ab. Fat** | **Liver** |
| --- | --- | --- | --- |
| Total SNPs Analyzed | 148,860 | 217,628 | 155,875 |
| Total Read Counts | 70,385,651 | 112,093,032 | 83,352,055 |
| Average Read Count | 472.83 | 515.07 | 534.74 |
| Range of Read Counts | 20-73134 | 20-68765 | 20-82966 |

**Supplemental Table S8.** Binning of all informative SNP read counts broken down by tissue. Bins refer to the total counts for a SNP and reflect the number of SNPs with those counts

|  | **Breast Muscle** | | **Abdominal Fat** | | **Liver** | |
| --- | --- | --- | --- | --- | --- | --- |
| **Bins** | **Counts** | **Percent** | **Counts** | **Percent** | **Counts** | **Percent** |
| Bin 20-200 | 87135 | 58.53% | 111401 | 51.19% | 88204 | 56.59% |
| Bin 200-400 | 24369 | 16.37% | 40583 | 18.65% | 27131 | 17.41% |
| Bin 400-600 | 12322 | 8.28% | 21632 | 9.94% | 13427 | 8.61% |
| Bin 600-800 | 6760 | 4.54% | 12287 | 5.65% | 7197 | 4.62% |
| Bin 800-1000 | 4232 | 2.84% | 7671 | 3.52% | 4235 | 2.72% |
| Bin 1000-1200 | 2644 | 1.78% | 5017 | 2.31% | 2836 | 1.82% |
| Bin 1200-1400 | 1900 | 1.28% | 3502 | 1.61% | 2100 | 1.35% |
| Bin >1400 | 9498 | 6.38% | 15535 | 7.14% | 10745 | 6.89% |

**Supplemental Figure S5.** Ensembl’s VEP summary statistics for significant SNPs for all three tissues. Downstream variants specifically refer to SNPs found within 5,000 bases of the end of the gene. Downstream SNPs most likely represent issues with the current genome annotation found in VEP and the average distance for downstream SNPs was found to be 1695.79 nt for breast muscle, 1775.12 nt for abdominal fat, and 1670.81 nt for liver. VEP also rounds-up result percentages to the whole number in the “Coding Consequences” graph, so some data maybe lost in visualization. A full breakdown of all the groups can be seen in Supplemental Tables S12-14

**Functional Enrichment Analysis of Variants**

Variants were examined to see if there was statistical change in the enrichment of certain types of variants based on its functional classification by Ensembl’s VEP tool [16] when comparing informative variants versus variants showing ASE. Specifically, the proportions of variants in the annotations categories of “Consequence” and “Impact” were compared using Fisher’s Exact Test [17]. and the false discovery rate controlled by Benjamini-Hochberg p-value adjustment method [18] generating a final adjusted p-value. All results of these analysis can be seen in Supplemental Tables S9-S14

**Supplemental Table S9.** Exact test analysis of breast muscle variants based on variant effect prediction for overall “impact” comparing the informative variants versus significant ASE variants. Sorted in order of adjusted p-value (smallest to largest)

| **Impact** | **Inform Counts** | **Total Info Variants** | **Inform Percent (%)** | **Sig Counts** | **Total Sig Variants** | **Sig Percent (%)** | **Fisher’s Exact Test p-value** | **BH Adj**  **p-value** |
| --- | --- | --- | --- | --- | --- | --- | --- | --- |
| LOW | 47105 | 148860 | 31.64 | 5807 | 20775 | 27.95 | 1.96E-27 | 7.86E-27 |
| MODIFIER | 89892 | 148860 | 60.39 | 13348 | 20775 | 64.25 | 7.16E-27 | 1.43E-26 |
| HIGH | 86 | 148860 | 0.06 | 30 | 20775 | 0.14 | 5.31E-05 | 7.09E-05 |
| MODERATE | 11777 | 148860 | 7.91 | 1590 | 20775 | 7.65 | 2.01E-01 | 2.01E-01 |

**Supplemental Table S10.** Exact test analysis of liver variants based on variant effect prediction for overall “impact” comparing the informative variants versus significant ASE variants. Sorted in order of adjusted p-value (smallest to largest)

| **Impact** | **Inform Counts** | **Total**  **Info Variants** | **Inform Percent (%)** | **Sig Counts** | **Total Sig Variants** | **Sig Percent (%)** | **Fisher’s Exact Test p-value** | **BH Adj**  **p-value** |
| --- | --- | --- | --- | --- | --- | --- | --- | --- |
| LOW | 47004 | 155875 | 30.15 | 5633 | 21916 | 25.7 | 1.52E-42 | 6.09E-42 |
| MODIFIER | 96340 | 155875 | 61.81 | 14308 | 21916 | 65.29 | 1.60E-23 | 3.19E-23 |
| MODERATE | 12442 | 155875 | 7.98 | 1946 | 21916 | 8.88 | 6.45E-06 | 8.60E-06 |
| HIGH | 89 | 155875 | 0.06 | 29 | 21916 | 0.13 | 2.18E-04 | 2.18E-04 |

**Supplemental Table S11.** Exact test analysis of abdominal fat variants based on variant effect prediction for overall “impact” comparing the informative variants versus significant ASE variants. Sorted in order of adjusted p-value (smallest to largest)

| **Impact** | **Inform Counts** | **Total Info Variants** | **Inform Percent (%)** | **Sig Counts** | **Total Sig Variants** | **Sig Percent (%)** | **Fisher’s Exact Test p-value** | **BH Adj**  **p-value** |
| --- | --- | --- | --- | --- | --- | --- | --- | --- |
| LOW | 61127 | 217628 | 28.09 | 6859 | 28190 | 24.33 | 4.90E-41 | 1.96E-40 |
| MODIFIER | 139466 | 217628 | 64.08 | 19176 | 28190 | 68.02 | 3.67E-39 | 7.35E-39 |
| HIGH | 113 | 217628 | 0.05 | 28 | 28190 | 0.1 | 3.38E-03 | 4.51E-03 |
| MODERATE | 16922 | 217628 | 7.78 | 2127 | 28190 | 7.55 | 1.77E-01 | 1.77E-01 |

**Supplemental Table S12.** Exact test analysis of breast muscle variants based on variant effect prediction for overall “consequence” comparing the informative variants versus significant ASE variants. Sorted in order of adjusted p-value (smallest to largest)

| **Consequence** | **Inform Counts** | **Total Info Variants** | **Inform Percent (%)** | **Sig Counts** | **Total Sig Variants** | **Sig Percent (%)** | **Fisher’s Exact Test p-value** | **BH Adj p-value** |
| --- | --- | --- | --- | --- | --- | --- | --- | --- |
| intergenic variant | 5695 | 148860 | 3.83 | 1291 | 20775 | 6.21 | 8.85E-53 | 2.39E-51 |
| intron variant | 11074 | 148860 | 7.44 | 2029 | 20775 | 9.77 | 3.90E-30 | 5.26E-29 |
| synonymous variant | 46391 | 148860 | 31.16 | 5693 | 20775 | 27.4 | 1.15E-28 | 1.03E-27 |
| downstream gene variant | 36205 | 148860 | 24.32 | 4441 | 20775 | 21.38 | 5.05E-21 | 3.41E-20 |
| non coding transcript exon variant | 802 | 148860 | 0.54 | 203 | 20775 | 0.98 | 8.41E-13 | 4.54E-12 |
| 3 prime UTR variant | 22747 | 148860 | 15.28 | 3395 | 20775 | 16.34 | 8.19E-05 | 3.68E-04 |
| intron variant,non coding transcript variant | 259 | 148860 | 0.17 | 63 | 20775 | 0.3 | 1.65E-04 | 6.36E-04 |
| splice acceptor variant | 17 | 148860 | 0.01 | 11 | 20775 | 0.05 | 2.68E-04 | 9.03E-04 |
| splice donor variant | 21 | 148860 | 0.01 | 10 | 20775 | 0.05 | 2.91E-03 | 8.72E-03 |
| splice region variant,intron variant | 102 | 148860 | 0.07 | 27 | 20775 | 0.13 | 4.55E-03 | 1.23E-02 |
| upstream gene variant | 11070 | 148860 | 7.44 | 1650 | 20775 | 7.94 | 1.01E-02 | 2.47E-02 |
| splice region variant,5 prime UTR variant | 18 | 148860 | 0.01 | 6 | 20775 | 0.03 | 6.48E-02 | 1.46E-01 |
| missense variant | 11655 | 148860 | 7.83 | 1567 | 20775 | 7.54 | 1.51E-01 | 3.13E-01 |
| missense variant,splice region variant | 122 | 148860 | 0.08 | 23 | 20775 | 0.11 | 2.03E-01 | 3.71E-01 |
| stop retained variant | 19 | 148860 | 0.01 | 5 | 20775 | 0.02 | 2.06E-01 | 3.71E-01 |
| start lost,synonymous variant | 1 | 148860 | 0 | 1 | 20775 | 0 | 2.30E-01 | 3.88E-01 |
| start lost | 6 | 148860 | 0 | 2 | 20775 | 0.01 | 2.56E-01 | 4.06E-01 |
| splice acceptor variant,non coding transcript variant | 2 | 148860 | 0 | 1 | 20775 | 0 | 3.24E-01 | 4.61E-01 |
| splice region variant,intron variant,non coding transcript variant | 2 | 148860 | 0 | 1 | 20775 | 0 | 3.24E-01 | 4.61E-01 |
| stop lost | 6 | 148860 | 0 | 1 | 20775 | 0 | 5.99E-01 | 8.09E-01 |
| 5 prime UTR variant | 2039 | 148860 | 1.37 | 276 | 20775 | 1.33 | 6.55E-01 | 8.24E-01 |
| splice region variant,synonymous variant | 562 | 148860 | 0.38 | 74 | 20775 | 0.36 | 6.72E-01 | 8.24E-01 |
| stop gained | 31 | 148860 | 0.02 | 4 | 20775 | 0.02 | 1.00E+00 | 1.00E+00 |
| splice region variant,3 prime UTR variant | 9 | 148860 | 0.01 | 1 | 20775 | 0 | 1.00E+00 | 1.00E+00 |
| non coding transcript variant | 1 | 148860 | 0 | 0 | 20775 | 0 | 1.00E+00 | 1.00E+00 |
| splice donor variant,non coding transcript variant | 2 | 148860 | 0 | 0 | 20775 | 0 | 1.00E+00 | 1.00E+00 |
| splice region variant,non coding transcript exon variant | 2 | 148860 | 0 | 0 | 20775 | 0 | 1.00E+00 | 1.00E+00 |

**Supplemental Table S13.** Exact test analysis of liver variants based on variant effect prediction for overall “consequence” comparing the informative variants versus significant ASE variants. Sorted in order of adjusted p-value (smallest to largest)

| **Consequence** | **Inform Counts** | **Total Info Variants** | **Inform Percent (%)** | **Sig Counts** | **Total Sig Variants** | **Sig Percent (%)** | **Fisher’s Exact Test p-value** | **BH Adj p-value** |
| --- | --- | --- | --- | --- | --- | --- | --- | --- |
| intergenic variant | 5771 | 155875 | 3.7 | 1308 | 21916 | 5.97 | 7.35E-52 | 1.91E-50 |
| synonymous variant | 46145 | 155875 | 29.6 | 5484 | 21916 | 25.02 | 1.57E-45 | 2.04E-44 |
| downstream gene variant | 34048 | 155875 | 21.84 | 4237 | 21916 | 19.33 | 1.23E-17 | 1.07E-16 |
| intron variant | 19073 | 155875 | 12.24 | 3064 | 21916 | 13.98 | 5.05E-13 | 3.28E-12 |
| 3 prime UTR variant | 22434 | 155875 | 14.39 | 3450 | 21916 | 15.74 | 1.46E-07 | 7.59E-07 |
| non coding transcript exon variant | 656 | 155875 | 0.42 | 150 | 21916 | 0.68 | 2.93E-07 | 1.27E-06 |
| intron variant,non coding transcript variant | 237 | 155875 | 0.15 | 69 | 21916 | 0.31 | 7.63E-07 | 2.84E-06 |
| missense variant | 12285 | 155875 | 7.88 | 1920 | 21916 | 8.76 | 8.82E-06 | 2.86E-05 |
| splice acceptor variant | 17 | 155875 | 0.01 | 10 | 21916 | 0.05 | 9.23E-04 | 2.67E-03 |
| stop lost | 6 | 155875 | 0 | 5 | 21916 | 0.02 | 6.90E-03 | 1.79E-02 |
| splice region variant,intron variant | 183 | 155875 | 0.12 | 41 | 21916 | 0.19 | 1.05E-02 | 2.49E-02 |
| start lost | 6 | 155875 | 0 | 4 | 21916 | 0.02 | 2.62E-02 | 5.68E-02 |
| stop retained variant | 18 | 155875 | 0.01 | 6 | 21916 | 0.03 | 1.09E-01 | 2.18E-01 |
| 5 prime UTR variant | 2335 | 155875 | 1.5 | 348 | 21916 | 1.59 | 3.01E-01 | 5.51E-01 |
| splice region variant,intron variant,non coding transcript variant | 2 | 155875 | 0 | 1 | 21916 | 0 | 3.26E-01 | 5.51E-01 |
| splice region variant,5 prime UTR variant | 18 | 155875 | 0.01 | 4 | 21916 | 0.02 | 3.39E-01 | 5.51E-01 |
| missense variant,splice region variant | 157 | 155875 | 0.1 | 26 | 21916 | 0.12 | 4.31E-01 | 6.59E-01 |
| splice region variant,synonymous variant | 627 | 155875 | 0.4 | 95 | 21916 | 0.43 | 4.96E-01 | 7.16E-01 |
| upstream gene variant | 11785 | 155875 | 7.56 | 1682 | 21916 | 7.67 | 5.49E-01 | 7.51E-01 |
| splice donor variant | 26 | 155875 | 0.02 | 5 | 21916 | 0.02 | 5.80E-01 | 7.54E-01 |
| splice region variant,3 prime UTR variant | 10 | 155875 | 0.01 | 2 | 21916 | 0.01 | 6.52E-01 | 8.07E-01 |
| stop gained | 31 | 155875 | 0.02 | 5 | 21916 | 0.02 | 7.98E-01 | 9.43E-01 |
| splice acceptor variant,non coding transcript variant | 2 | 155875 | 0 | 0 | 21916 | 0 | 1.00E+00 | 1.00E+00 |
| coding sequence variant | 1 | 155875 | 0 | 0 | 21916 | 0 | 1.00E+00 | 1.00E+00 |
| splice donor variant,non coding transcript variant | 1 | 155875 | 0 | 0 | 21916 | 0 | 1.00E+00 | 1.00E+00 |
| splice region variant,non coding transcript exon variant | 1 | 155875 | 0 | 0 | 21916 | 0 | 1.00E+00 | 1.00E+00 |

**Supplemental Table S14.** Exact test analysis of abdominal fat variants based on variant effect prediction for overall “consequence” comparing the informative variants versus significant ASE variants. Sorted in order of adjusted p-value (smallest to largest).

| **Consequence** | **Inform Counts** | **Total Info Variants** | **Inform Percent (%)** | **Sig Counts** | **Total Sig Variants** | **Sig Percent (%)** | **Fisher’s Exact Test p-value** | **BH Adj p-value** |
| --- | --- | --- | --- | --- | --- | --- | --- | --- |
| intergenic variant | 9246 | 217628 | 4.25 | 1920 | 28190 | 6.81 | 4.43E-75 | 1.24E-73 |
| synonymous variant | 59923 | 217628 | 27.53 | 6673 | 28190 | 23.67 | 6.81E-44 | 9.53E-43 |
| intron variant,non coding transcript variant | 770 | 217628 | 0.35 | 246 | 28190 | 0.87 | 6.23E-30 | 5.82E-29 |
| 3 prime UTR variant | 28447 | 217628 | 13.07 | 4350 | 28190 | 15.43 | 4.94E-27 | 3.46E-26 |
| non coding transcript exon variant | 1160 | 217628 | 0.53 | 263 | 28190 | 0.93 | 1.04E-14 | 5.85E-14 |
| downstream gene variant | 52305 | 217628 | 24.03 | 6349 | 28190 | 22.52 | 1.81E-08 | 8.45E-08 |
| intron variant | 26509 | 217628 | 12.18 | 3158 | 28190 | 11.2 | 1.75E-06 | 6.99E-06 |
| upstream gene variant | 17836 | 217628 | 8.2 | 2462 | 28190 | 8.73 | 2.14E-03 | 7.48E-03 |
| splice region variant,intron variant | 224 | 217628 | 0.1 | 47 | 28190 | 0.17 | 4.02E-03 | 1.25E-02 |
| splice donor variant | 28 | 217628 | 0.01 | 10 | 28190 | 0.04 | 8.95E-03 | 2.51E-02 |
| splice region variant,intron variant,non coding transcript variant | 3 | 217628 | 0 | 2 | 28190 | 0.01 | 1.04E-01 | 2.61E-01 |
| splice acceptor variant | 20 | 217628 | 0.01 | 6 | 28190 | 0.02 | 1.12E-01 | 2.61E-01 |
| splice region variant,5 prime UTR variant | 33 | 217628 | 0.02 | 8 | 28190 | 0.03 | 1.34E-01 | 2.88E-01 |
| missense variant | 16696 | 217628 | 7.67 | 2095 | 28190 | 7.43 | 1.56E-01 | 3.13E-01 |
| splice region variant,non coding transcript exon variant | 5 | 217628 | 0 | 2 | 28190 | 0.01 | 1.87E-01 | 3.49E-01 |
| stop lost | 8 | 217628 | 0 | 2 | 28190 | 0.01 | 3.21E-01 | 5.62E-01 |
| stop gained | 43 | 217628 | 0.02 | 8 | 28190 | 0.03 | 3.74E-01 | 6.17E-01 |
| 5 prime UTR variant | 3190 | 217628 | 1.47 | 428 | 28190 | 1.52 | 4.94E-01 | 7.69E-01 |
| stop retained variant | 23 | 217628 | 0.01 | 4 | 28190 | 0.01 | 5.43E-01 | 8.00E-01 |
| missense variant,splice region variant | 226 | 217628 | 0.1 | 32 | 28190 | 0.11 | 6.25E-01 | 8.75E-01 |
| start lost | 12 | 217628 | 0.01 | 2 | 28190 | 0.01 | 6.70E-01 | 8.75E-01 |
| splice region variant,3 prime UTR variant | 13 | 217628 | 0.01 | 2 | 28190 | 0.01 | 6.87E-01 | 8.75E-01 |
| splice region variant,synonymous variant | 903 | 217628 | 0.41 | 121 | 28190 | 0.43 | 7.31E-01 | 8.90E-01 |
| non coding transcript variant | 1 | 217628 | 0 | 0 | 28190 | 0 | 1.00E+00 | 1.00E+00 |
| splice acceptor variant,non coding transcript variant | 1 | 217628 | 0 | 0 | 28190 | 0 | 1.00E+00 | 1.00E+00 |
| coding sequence variant | 1 | 217628 | 0 | 0 | 28190 | 0 | 1.00E+00 | 1.00E+00 |
| stop gained,splice region variant | 1 | 217628 | 0 | 0 | 28190 | 0 | 1.00E+00 | 1.00E+00 |
| mature miRNA variant | 1 | 217628 | 0 | 0 | 28190 | 0 | 1.00E+00 | 1.00E+00 |

**Supplemental Figure S6.** Venn diagram comparing overlapping genes among the three tissues. On left are genes identified from the informative SNPs using VEP and on right are genes identified from significant SNPs using VEP.

**Expanded View of DAVID Results**

Significant ASE SNPs identified using VADT were uploaded to Ensembl’s VEP [16] and results were parsed using a custom script to identify all unique Ensembl gene IDs, which were separated into various categories based on how they overlapped in Supplemental Figure S5. and results uploaded to DAVID [19, 20]. The results of the analysis can be seen in Figure 5., and an expanded view of the pathway enrichment results from DAVID can be seen in Supplemental Tables S15-19.

**Supplemental Table S15.** Breast Muscle tissue results from DAVID, 1319 Ensembl IDs submitted and 817 Ensembl IDs matched for analysis. A total of three clusters showed statistical significance (FDR p-value <0.1 and Enrichment Score >1.3) and only significant terms listed.

| **Annotation Cluster 1** | **Enrichment Score: 2.89** | **Count** | **P_Value** | **Benjamini** |
| --- | --- | --- | --- | --- |
| UP_KEYWORDS | Zinc-finger | 42 | 5.00E-04 | 5.50E-02 |
| UP_KEYWORDS | Zinc | 56 | 9.00E-04 | 6.60E-02 |
| **Annotation Cluster 3** | **Enrichment Score: 2.3** | **Count** | **P_Value** | **Benjamini** |
| GOTERM_CC_DIRECT | Cul3-RING ubiquitin ligase complex | 12 | 4.50E-04 | 4.80E-02 |
| **Annotation Cluster 5** | **Enrichment Score: 1.75** | **Count** | **P_Value** | **Benjamini** |
| GOTERM_BP_DIRECT | ubiquitin-dependent protein catabolic process | 17 | 5.00E-05 | 8.30E-02 |

**Supplemental Table S16.** Breast Muscle/Liver tissue results from DAVID, 430 Ensembl IDs submitted and 281 Ensembl IDs matched for analysis. Only annotation cluster 1 showed statistical significance (FDR p-value <0.1 and Enrichment Score >1.3) and only significant terms listed in table.

| **Annotation Cluster 1** | **Enrichment Score: 3.15** | **Count** | **P_Value** | **Benjamini** |
| --- | --- | --- | --- | --- |
| KEGG_PATHWAY | Biosynthesis of antibiotics | 15 | 3.50E-04 | 3.50E-02 |
| KEGG_PATHWAY | Biosynthesis of amino acids | 8 | 9.60E-04 | 4.80E-02 |
| KEGG_PATHWAY | Carbon metabolism | 10 | 1.00E-03 | 3.50E-02 |

**Supplemental Table S17.** Breast Muscle/Abdominal Fat tissue results from DAVID, 1320 Ensembl IDs submitted and 833 Ensembl IDs matched for enrichment. Only annotation cluster 1 showed significance (FDR p-value <0.1 and Enrichment Score >1.3) and only significant terms listed in table.

| **Annotation Cluster 1** | **Enrichment Score: 2.85** | **Count** | **P_Value** | **Benjamini** |
| --- | --- | --- | --- | --- |
| INTERPRO | Collagen triple helix repeat | 14 | 2.30E-05 | 2.90E-02 |
| UP_KEYWORDS | Collagen | 11 | 1.10E-04 | 2.50E-02 |
| GOTERM_CC_DIRECT | collagen trimer | 10 | 2.30E-04 | 4.80E-02 |

**Supplemental Table S18.** All three tissue results from DAVID, 1715 Ensembl IDs submitted and 1036 Ensembl IDs matched for enrichment. A total of five clusters showed significance (FDR p-value <0.1 and Enrichment Score >1.3). and only significant terms listed in table.

| **Annotation Cluster 1** | **Enrichment Score: 8.27** | **Count** | **P_Value** | **Benjamini** |
| --- | --- | --- | --- | --- |
| GOTERM_CC_DIRECT | cytosolic large ribosomal subunit | 21 | 2.50E-10 | 3.20E-08 |
| GOTERM_MF_DIRECT | structural constituent of ribosome | 37 | 4.60E-10 | 3.30E-07 |
| KEGG_PATHWAY | Ribosome | 37 | 9.60E-10 | 1.40E-07 |
| UP_KEYWORDS | Ribosomal protein | 24 | 2.40E-09 | 6.90E-07 |
| UP_KEYWORDS | Ribonucleoprotein | 25 | 5.10E-08 | 7.40E-06 |
| GOTERM_BP_DIRECT | translation | 29 | 1.80E-06 | 2.00E-03 |
| **Annotation Cluster 2** | **Enrichment Score: 2.97** | **Count** | **P_Value** | **Benjamini** |
| INTERPRO | Glutathione S-transferase, C-terminal-like | 12 | 3.50E-06 | 3.00E-03 |
| INTERPRO | Glutathione S-transferase, C-terminal | 8 | 7.90E-05 | 4.40E-02 |
| GOTERM_BP_DIRECT | glutathione metabolic process | 9 | 8.80E-05 | 3.80E-02 |
| GOTERM_MF_DIRECT | glutathione transferase activity | 8 | 2.90E-04 | 6.70E-02 |
| KEGG_PATHWAY | Glutathione metabolism | 11 | 4.40E-03 | 5.20E-02 |
| KEGG_PATHWAY | Metabolism of xenobiotics by cytochrome P450 | 9 | 1.20E-02 | 9.30E-02 |
| **Annotation Cluster 3** | **Enrichment Score: 2.65** | **Count** | **P_Value** | **Benjamini** |
| KEGG_PATHWAY | Fatty acid degradation | 16 | 2.60E-07 | 1.90E-05 |
| KEGG_PATHWAY | Valine, leucine and isoleucine degradation | 14 | 3.60E-04 | 8.80E-03 |
| KEGG_PATHWAY | beta-Alanine metabolism | 10 | 1.10E-03 | 2.30E-02 |
| KEGG_PATHWAY | Pyruvate metabolism | 11 | 1.80E-03 | 2.90E-02 |
| KEGG_PATHWAY | Arginine and proline metabolism | 12 | 2.40E-03 | 3.50E-02 |
| **Annotation Cluster 4** | **Enrichment Score: 1.93** | **Count** | **P_Value** | **Benjamini** |
| KEGG_PATHWAY | Glycolysis / Gluconeogenesis | 14 | 1.70E-03 | 3.00E-02 |
| **Annotation Cluster 7** | **Enrichment Score: 1.54** | **Count** | **P_Value** | **Benjamini** |
| KEGG_PATHWAY | Butanoate metabolism | 8 | 9.20E-03 | 8.00E-02 |
| KEGG_PATHWAY | Propanoate metabolism | 8 | 1.20E-02 | 9.50E-02 |

**Supplemental Table S19.** Liver tissue results from DAVID, 1057 Ensembl IDs submitted and 703 Ensembl IDs matched for enrichment. Only 2 annotations clusters visualized, however a total of 7 clusters had significance (FDR p-value <0.1 and Enrichment Score >1.3) and only significant terms listed in table.

| **Annotation Cluster 1** | **Enrichment Score: 4.25** | **Count** | **P_Value** | **Benjamini** |
| --- | --- | --- | --- | --- |
| GOTERM_BP_DIRECT | fibrinolysis | 8 | 1.40E-07 | 2.40E-04 |
| SMART | Tryp SPc | 14 | 1.60E-07 | 3.90E-05 |
| INTERPRO | Peptidase S1A, chymotrypsin-type | 14 | 2.80E-07 | 3.30E-04 |
| UP_KEYWORDS | Hemostasis | 7 | 5.60E-07 | 1.40E-04 |
| UP_KEYWORDS | Blood coagulation | 7 | 5.60E-07 | 1.40E-04 |
| INTERPRO | Peptidase S1 | 14 | 1.10E-06 | 6.60E-04 |
| INTERPRO | Trypsin-like cysteine/serine peptidase domain | 14 | 2.30E-06 | 9.20E-04 |
| UP_KEYWORDS | Serine protease | 13 | 4.20E-06 | 5.40E-04 |
| GOTERM_MF_DIRECT | serine-type endopeptidase activity | 16 | 1.70E-05 | 9.40E-03 |
| INTERPRO | Peptidase S1, trypsin family, active site | 10 | 2.00E-04 | 2.40E-02 |
| UP_KEYWORDS | Protease | 20 | 3.90E-03 | 9.50E-02 |
| **Annotation Cluster 2** | **Enrichment Score: 3.75** | **Count** | **P_Value** | **Benjamini** |
| INTERPRO | Cytochrome P450 | 11 | 2.80E-06 | 8.30E-04 |
| GOTERM_MF_DIRECT | iron ion binding | 18 | 2.20E-05 | 6.20E-03 |
| COG_ONTOLOGY | Secondary metabolites biosynthesis, transport, and catabolism | 14 | 2.70E-05 | 7.30E-04 |
| COG_ONTOLOGY | Secondary metabolites biosynthesis, transport, and catabolism | 14 | 2.70E-05 | 7.30E-04 |
| COG_ONTOLOGY | Secondary metabolites biosynthesis, transport, and catabolism | 14 | 2.70E-05 | 7.30E-04 |
| UP_KEYWORDS | Iron | 18 | 1.60E-04 | 1.30E-02 |
| GOTERM_MF_DIRECT | heme binding | 15 | 2.00E-04 | 3.60E-02 |
| UP_KEYWORDS | Monooxygenase | 9 | 8.60E-04 | 4.30E-02 |
| UP_KEYWORDS | Heme | 11 | 9.80E-04 | 4.10E-02 |
| UP_KEYWORDS | Oxidoreductase | 23 | 1.10E-03 | 3.80E-02 |

**Top ASE Candidate Genes**

**Supplemental Table S20.** Genes with the strongest ASE evidence identified using VADT, VEP and normalizing gene’s ASE SNP count using the following equation: (gene’s ASE SNP count/ gene’s informative SNP count) * 100. Genes were considered as showing the strongest ASE evidence if the average normalization value among all three tissues (breast muscle, abdominal fat and liver) was >=80%.

| No. | Ensembl Gene ID | Gene Symbol | Avg. Normalized |
| --- | --- | --- | --- |
| 1 | ENSGALG00000038687 | - | 100.00 |
| 2 | ENSGALG00000021139 | IGLL1 | 100.00 |
| 3 | ENSGALG00000042449 | - | 100.00 |
| 4 | ENSGALG00000039383 | - | 100.00 |
| 5 | ENSGALG00000041885 | - | 100.00 |
| 6 | ENSGALG00000029837 | OST4 | 100.00 |
| 7 | ENSGALG00000029729 | - | 100.00 |
| 8 | ENSGALG00000035138 | UBL5 | 100.00 |
| 9 | ENSGALG00000043742 | - | 100.00 |
| 10 | ENSGALG00000032272 | - | 100.00 |
| 11 | ENSGALG00000007611 | RPL35A | 100.00 |
| 12 | ENSGALG00000008066 | UQCR10 | 100.00 |
| 13 | ENSGALG00000037876 | - | 100.00 |
| 14 | ENSGALG00000039867 | - | 100.00 |
| 15 | ENSGALG00000034352 | MRPL43 | 100.00 |
| 16 | ENSGALG00000041562 | - | 100.00 |
| 17 | ENSGALG00000034151 | - | 100.00 |
| 18 | ENSGALG00000012229 | RPS29 | 100.00 |
| 19 | ENSGALG00000042642 | RARRES2 | 100.00 |
| 20 | ENSGALG00000012299 | - | 100.00 |
| 21 | ENSGALG00000046114 | - | 100.00 |
| 22 | ENSGALG00000021365 | DCTN3 | 100.00 |
| 23 | ENSGALG00000043768 | ND2 | 100.00 |
| 24 | ENSGALG00000005490 | RPS2 | 94.44 |
| 25 | ENSGALG00000041793 | RF00004 | 94.44 |
| 26 | ENSGALG00000014585 | - | 94.44 |
| 27 | ENSGALG00000040260 | TUBA1C | 93.33 |
| 28 | ENSGALG00000041380 | BF2 | 92.70 |
| 29 | ENSGALG00000040576 | - | 92.59 |
| 30 | ENSGALG00000022871 | HHLA2 | 91.85 |
| 31 | ENSGALG00000037783 | - | 91.67 |
| 32 | ENSGALG00000042863 | SMIM4 | 91.67 |
| 33 | ENSGALG00000043565 | - | 91.67 |
| 34 | ENSGALG00000010811 | LGMN | 90.91 |
| 35 | ENSGALG00000041597 | - | 90.48 |
| 36 | ENSGALG00000040628 | - | 90.48 |
| 37 | ENSGALG00000031403 | - | 88.89 |
| 38 | ENSGALG00000030109 | - | 88.89 |
| 39 | ENSGALG00000036158 | - | 88.89 |
| 40 | ENSGALG00000001529 | THYN1 | 88.89 |
| 41 | ENSGALG00000032757 | - | 88.89 |
| 42 | ENSGALG00000036173 | - | 88.89 |
| 43 | ENSGALG00000012488 | TST | 87.78 |
| 44 | ENSGALG00000033672 | - | 87.63 |
| 45 | ENSGALG00000004028 | - | 87.50 |
| 46 | ENSGALG00000039881 | - | 87.42 |
| 47 | ENSGALG00000038410 | - | 87.18 |
| 48 | ENSGALG00000034721 | - | 86.90 |
| 49 | ENSGALG00000033302 | - | 85.93 |
| 50 | ENSGALG00000044239 | - | 84.85 |
| 51 | ENSGALG00000033932 | BF1 | 84.52 |
| 52 | ENSGALG00000037405 | - | 83.33 |
| 53 | ENSGALG00000039548 | - | 83.33 |
| 54 | ENSGALG00000031701 | - | 83.33 |
| 55 | ENSGALG00000036214 | PARK7 | 83.33 |
| 56 | ENSGALG00000027483 | GLRX | 83.33 |
| 57 | ENSGALG00000030940 | BLB2 | 83.33 |
| 58 | ENSGALG00000031865 | - | 83.33 |
| 59 | ENSGALG00000045034 | - | 83.33 |
| 60 | ENSGALG00000046185 | - | 83.33 |
| 61 | ENSGALG00000005849 | PPDPF | 83.33 |
| 62 | ENSGALG00000028520 | CST3 | 83.33 |
| 63 | ENSGALG00000032142 | COX1 | 83.33 |
| 64 | ENSGALG00000036145 | - | 83.33 |
| 65 | ENSGALG00000001330 | ATP5MC1 | 83.33 |
| 66 | ENSGALG00000004521 | GPX3 | 82.58 |
| 67 | ENSGALG00000004769 | PSAP | 82.38 |
| 68 | ENSGALG00000001718 | CCNG1 | 82.25 |
| 69 | ENSGALG00000030189 | - | 82.14 |
| 70 | ENSGALG00000026152 | GBP | 81.93 |
| 71 | ENSGALG00000000293 | - | 81.68 |
| 72 | ENSGALG00000032653 | - | 81.24 |
| 73 | ENSGALG00000012119 | MARCO | 81.15 |
| 74 | ENSGALG00000037953 | TUBA1A | 80.64 |
| 75 | ENSGALG00000029339 | - | 80.56 |
| 76 | ENSGALG00000033116 | CLC2DL3 | 80.44 |
| 77 | ENSGALG00000040068 | - | 80.11 |
| 78 | ENSGALG00000007403 | PEBP1 | 80.00 |

**Examination of Feed Efficiency Status (HFE vs LFE) and ASE**

To investigate whether ASE was influenced by the feed efficiency status of the samples (HFE vs LFE) we implemented a “Fisher’s Exact Test,” comparing the two groups [17]. This analysis was performed using a custom developed python script that mined the meta-data and ASE results from VADT and implemented the Fisher’s Exact Test for 2x2 contingency table using SciPy [21] and the resulting p-values adjusted using the Benjamini-Hochberg method [18]. An example of the Fisher’s Exact Test implemented comparing HFE vs. LFE can be seen in Supplemental Table S21 (note: the final adjusted p-value was based on all the variants tested). The code implemented to perform this analysis is available on GitHub at the following link (<https://github.com/mjtiv/HFEvsLFE_FisherExactTest>). The specifics of the analysis will be discussed in much greater details with overall findings.

**Supplemental Table S21.** Example of the Fish’s Exact Test comparing HFE vs. LFE samples.

| Breast Muscle Samples | | |
| --- | --- | --- |
| chr18:592905     rs740024577 | | |
|  | HFE | LFE |
| Significant ASE | 4 | 0 |
| Non-Sig. Biallelic | 1 | 7 |
|  | | |
| p-value = 0.0101 | | |
| adjusted p-value = 1.00 | | |

Specifically, in this study we utilized the VADT output results from multi-dimensional FDR-controlling analysis which extracted all the variants with at least one sample showing statistical significance for ASE. We then examined the data at various filtering cutoffs based on the number of biallelic samples in each group. Meaning both the HFE and LFE groups need to contain a minimal number of biallelic samples to be tested using Fisher’s Exact Test. For example, in Table RRII 2, this variant would be found in the minimal samples groups 2-5 because it has a total of 5 biallelic samples in the HFE group (4 ASE + 1 Non. Sig. Biallelic) and 7 biallelic samples in the LFE group with the HFE group being the limiting factor. Now the various results of these sample minimum cutoffs can be seen in Supplemental Table S22. As shown each tissue has a different sample cutoff where the most significant p-value is identified. But, no matter what biallelic sample minimal cutoff was applied (reduction of overall data dimensionality aka number of variants tested) no adjusted p-value reach significance. Meaning our power of detection for comparing HFE vs LFE for ASE enrichment is unpowered by the number of sample and the results more exploratory.

Examining Supplemental Table S22 each tissue has a different inflection point for where the smallest p-value is found with lowest number of variants tests (highlighted yellow). The average sample minimum for the HFE vs. LFE biallelic groups inflection point was being found to be 5.66. So, a minimal sample number of 5 was selected for all three tissues and the most significant variants based on Fisher’s Exact test extracted (Supplemental Table S23). As seen in the table exploratory biological insights can start to be identified between the two groups, but caution needs to be taken because no variant reached statistical significance after p-value adjustment using Benjamini-Hochberg method [18].

We also tried to explore a different statistical model to see if that may help the statistical power issue. Specifically, we implemented a Z-test for comparing two proportions and the final p-values adjusted using the Benjamini-Hochberg method. The code implemented can be found at the following github (<https://github.com/mjtiv/proportional_hypothesis_test>). However, this model encountered the same limitations as Fisher’s Exact Test and no variants reached significance after p-value adjustments.

In summary there appears to be strong relationship between feed efficiency status (HFE & LFE) and ASE, however, due to the dimensionality of the data we are unpowered in this study and all results more exploratory and speculative.

**Supplemental Table S22.** Most Significant p-value at various minimal biallelic sample cutoffs with corresponding adjusted p-value for the various tissues. P-values were determined using “Fisher’s Exact Test” and adjusted using Bejamini Hochberg method [18].

| **Breast Muscle** | | | | | | | | | |
| --- | --- | --- | --- | --- | --- | --- | --- | --- | --- |
| **Minimum Biallelic Samples in Each Group** | **2** | **3** | **4** | **5** | **6** | **7** | **8** | **9** | **10** |
| p-value | 0.0101 | 0.0101 | 0.0101 | 0.0101 | 0.0210 | 0.0210 | 0.0325 | 0.0325 | 0.0573 |
| adj. p-value | 1.0000 | 1.0000 | 1.0000 | 1.0000 | 1.0000 | 1.0000 | 1.0000 | 1.0000 | 1.0000 |
| Total Variants (n) | 9906 | 7459 | 5234 | 3349 | 1813 | 789 | 356 | 141 | 54 |
|  | | | | | | | | | |
| **Abdominal Fat** | | | | | | | | | |
| **Minimum Biallelic Samples in Each Group** | **2** | **3** | **4** | **5** | **6** | **7** | **8** | **9** | **10** |
| p-value | 0.0011 | 0.0011 | 0.0011 | 0.0011 | 0.0011 | 0.0011 | 0.0011 | 0.0244 | 0.0698 |
| adj. p-value | 1.0000 | 1.0000 | 1.0000 | 1.0000 | 1.0000 | 1.0000 | 0.4478 | 1.0000 | 1.0000 |
| Total Variants (n) | 13152 | 10009 | 7096 | 4517 | 2345 | 1029 | 426 | 169 | 73 |
|  | | | | | | | | | |
| **Liver** | | | | | | | | | |
| **Minimum Biallelic Samples in Each Group** | **2** | **3** | **4** | **5** | **6** | **7** | **8** | **9** | **10** |
| p-value | 0.0079 | 0.0079 | 0.0079 | 0.0091 | 0.0091 | 0.0091 | 0.0091 | 0.0091 | 0.0237 |
| adj. p-value | 1.0000 | 1.0000 | 1.0000 | 1.0000 | 1.0000 | 1.0000 | 1.0000 | 1.0000 | 1.0000 |
| Total Variants (n) | 11015 | 8502 | 6319 | 4174 | 2381 | 1170 | 473 | 199 | 87 |

**Supplemental Table S23.** Top Ten Most Significant p-values from the various tissues analyzed using the “Fisher Exact Test”, all results should be considered exploratory because after adjustment no statistical significance was found. All variants were annotated with Ensembl’s VEP tool [16].

| **Breast Muscle** | | | | | | | | | | | | | | | | | | |
| --- | --- | --- | --- | --- | --- | --- | --- | --- | --- | --- | --- | --- | --- | --- | --- | --- | --- | --- |
| Chrom | Pos | ID | Symbol | Consequence | Impact | Ref | Alt | Total Biallelic | Total ASE | ASE Alleles | ASE Alleles HFE | ASE Alleles LFE | Sig ASE HFE | NonSig HFE Biallelic | Sig ASE LFE | NonSig LFE Biallelic | FisherExactTest Pvalue | BH Adj Pvalue |
| 18 | 592905 | rs740024577 | MYH1E | synonymous variant | LOW | G | C | 12 | 4 | Alt,Ref | Alt,Ref | NaN | 4 | 1 | 0 | 7 | 0.0101 | 1.00 |
| 1 | 48055366 | rs3137407 | MGP | 5 prime UTR variant | MODIFIER | A | G | 12 | 6 | Alt,Ref | Alt,Ref | Ref | 5 | 0 | 1 | 6 | 0.0152 | 1.00 |
| 6 | 11945053 | rs731623904 | PSAP | downstream gene variant | MODIFIER | A | T | 15 | 5 | Alt,Ref | Alt,Ref | Alt | 4 | 1 | 1 | 9 | 0.0170 | 1.00 |
| 15 | 6272384 | rs316198758 | - | 3 prime UTR variant | MODIFIER | C | T | 13 | 4 | Alt | NaN | Alt | 0 | 7 | 4 | 2 | 0.0210 | 1.00 |
| 1 | 1.96E+08 | rs741034191 | UCP3 | 3 prime UTR variant | MODIFIER | T | C | 14 | 9 | Alt,Ref | Alt | Alt,Ref | 2 | 5 | 7 | 0 | 0.0210 | 1.00 |
| 4 | 60451329 | rs317713926 | - | intergenic variant | MODIFIER | G | A | 14 | 9 | Ref | Ref | Ref | 2 | 5 | 7 | 0 | 0.0210 | 1.00 |
| 14 | 15535716 | rs315815936 | - | splice region variant | LOW | C | T | 17 | 14 | Ref | Ref | Ref | 3 | 3 | 11 | 0 | 0.0294 | 1.00 |
| 15 | 7980859 | . | IGLL1 | upstream gene variant | MODIFIER | G | T | 13 | 8 | Alt | Alt | Alt | 7 | 1 | 1 | 4 | 0.0319 | 1.00 |
| 18 | 648111 | . | MYH1C | synonymous variant | LOW | A | G | 19 | 15 | Alt,Ref | Alt | Alt,Ref | 5 | 4 | 10 | 0 | 0.0325 | 1.00 |
| 7 | 35421795 | rs732198551 | - | upstream gene variant | MODIFIER | C | T | 17 | 7 | Alt,Ref | NaN | Alt,Ref | 0 | 6 | 7 | 4 | 0.0345 | 1.00 |
|  | | | | | | | | | | | | | | | | | | |
| **Abdominal Fat** | | | | | | | | | | | | | | | | | | |
| Chrom | Pos | ID | Symbol | Consequence | Impact | Ref | Alt | Total Biallelic | Total ASE | ASE Alleles | ASE Alleles HFE | ASE Alleles LFE | Sig ASE HFE | NonSig HFE Biallelic | Sig ASE LFE | NonSig LFE Biallelic | FisherExactTest Pvalue | BH Adj Pvalue |
| 6 | 17492385 | rs316181561 | SCD | downstream gene variant | MODIFIER | G | A | 18 | 8 | Alt,Ref | NaN | Alt,Ref | 0 | 8 | 8 | 2 | 0.0011 | 1.00 |
| 6 | 17799268 | rs314959319 | WASHC2C | synonymous variant | LOW | C | T | 12 | 4 | Alt,Ref | NaN | Alt,Ref | 0 | 7 | 4 | 1 | 0.0101 | 1.00 |
| 2 | 1.3E+08 | rs741701432 | FZD6 | synonymous variant | LOW | T | C | 14 | 4 | Alt | NaN | Alt | 0 | 8 | 4 | 2 | 0.0150 | 1.00 |
| 25 | 2498523 | rs317661666 | PRPF3 | intron variant | MODIFIER | C | T | 14 | 10 | Alt | Alt | Alt | 2 | 4 | 8 | 0 | 0.0150 | 1.00 |
| 6 | 17829489 | rs316057925 | - | intron variant | MODIFIER | G | A | 11 | 5 | Ref | NaN | Ref | 0 | 5 | 5 | 1 | 0.0152 | 1.00 |
| 12 | 8953422 | rs738059649 | ABHD6 | 3 prime UTR variant | MODIFIER | G | A | 11 | 4 | Alt | NaN | Alt | 0 | 6 | 4 | 1 | 0.0152 | 1.00 |
| 18 | 9069305 | rs733111794 | COG1 | upstream gene variant | MODIFIER | G | A | 13 | 9 | Ref,Alt | Ref,Alt | Alt,Ref | 2 | 4 | 7 | 0 | 0.0210 | 1.00 |
| AADN04024019.1 | 1950 | . | - | intergenic variant | MODIFIER | T | A | 13 | 8 | Alt | Alt | Alt | 2 | 5 | 6 | 0 | 0.0210 | 1.00 |
| 1 | 62179209 | rs313465617 | AKR1B10L1 | synonymous variant | LOW | C | T | 21 | 9 | Alt,Ref | Alt | Alt,Ref | 1 | 8 | 8 | 4 | 0.0244 | 1.00 |
| 4 | 14152041 | rs312816228 | COL4A6 | downstream gene variant | MODIFIER | A | T | 15 | 4 | Alt | Alt | NaN | 4 | 3 | 0 | 8 | 0.0256 | 1.00 |
|  | | | | | | | | | | | | | | | | | | |
| **Liver** | | | | | | | | | | | | | | | | | | |
| Chrom | Pos | ID | Symbol | Consequence | Impact | Ref | Alt | Total Biallelic | Total ASE | ASE Alleles | ASE Alleles HFE | ASE Alleles LFE | Sig ASE HFE | NonSig HFE Biallelic | Sig ASE LFE | NonSig LFE Biallelic | FisherExactTest Pvalue | BH Adj Pvalue |
| 27 | 3715208 | rs741197236 | CALCOCO2 | missense variant | MODERATE | G | C | 18 | 6 | Ref | Ref | NaN | 6 | 3 | 0 | 9 | 0.0090 | 1.00 |
| 3 | 1.1E+08 | rs738402953 | CYP39A1 | downstream gene variant | MODIFIER | T | C | 12 | 4 | Ref | NaN | Ref | 0 | 7 | 4 | 1 | 0.0101 | 1.00 |
| Z | 12979182 | rs737532583 | C6 | missense variant | MODERATE | T | C | 19 | 13 | Ref | Ref | Ref | 9 | 0 | 4 | 6 | 0.0108 | 1.00 |
| AADN04023156.1 | 1481 | . | - | intergenic variant | MODIFIER | A | G | 20 | 13 | Alt,Ref | Alt,Ref | Alt,Ref | 8 | 0 | 5 | 7 | 0.0147 | 1.00 |
| 6 | 17589583 | rs732575111 | CYP2C23b | missense variant | MODERATE | T | G | 11 | 7 | Alt,Ref | Alt,Ref | Alt | 6 | 0 | 1 | 4 | 0.0152 | 1.00 |
| 12 | 828323 | rs314966458 | - | missense variant | MODERATE | G | A | 18 | 9 | Alt,Ref | Ref | Alt,Ref | 1 | 7 | 8 | 2 | 0.0152 | 1.00 |
| 4 | 52135542 | rs731108705 | SULT1B | synonymous variant | LOW | G | A | 21 | 14 | Alt | Alt | Alt | 10 | 1 | 4 | 6 | 0.0237 | 1.00 |
| 2 | 1.09E+08 | rs80621109 | C2H8ORF22 | 3 prime UTR variant | MODIFIER | T | C | 16 | 5 | Ref | Ref | NaN | 5 | 3 | 0 | 8 | 0.0256 | 1.00 |
| 6 | 15118799 | rs10731873 | ADK | synonymous variant | LOW | A | G | 14 | 3 | Ref | Ref | NaN | 3 | 2 | 0 | 9 | 0.0275 | 1.00 |
| 27 | 1855007 | rs314042055 | - | missense variant | MODERATE | G | T | 14 | 3 | Alt | Alt | NaN | 3 | 2 | 0 | 9 | 0.0275 | 1.00 |

**Work Cited**

1. Zhuo, Z., et al., *RNA-Seq Analysis of Abdominal Fat Reveals Differences between Modern Commercial Broiler Chickens with High and Low Feed Efficiencies.* PLoS One, 2015. **10**(8): p. e0135810.

2. Zhou, N., W.R. Lee, and B. Abasht, *Messenger RNA sequencing and pathway analysis provide novel insights into the biological basis of chickens' feed efficiency.* BMC Genomics, 2015. **16**: p. 195.

3. GATK. *Calling Variants in RNA-seq*. 2014 2015-12-07 [cited 2016 09-29]; Available from: <https://software.broadinstitute.org/gatk/guide/article?id=3891>.

4. Dobin, A., *STAR Manual 2.5.1a*, C.S. Harbors, Editor. 2016: <https://github.com/alexdobin/STAR/blob/master/doc/STARmanual.pdf>.

5. Dobin, A., et al., *STAR: Ultrafast Universal RNA-Seq Aligner.* Bioinformatics, 2012. **29**(1): p. 15-21.

6. ENSEMBL, *Genome assembly: Gallus_gallus-5.0*, ENSEMBL, Editor. 2016: [www.ensembl.org](file:///D:\Dropbox\Abasht_Lab_Folder_Joe_Tomlinson\ASE_PROJECT_EVERYTHING\VADT_PAPER_ALL_DOCUMENTS\FINAL_Paper_Submission\Supplemental_Sections\www.ensembl.org).

7. Zerbino, D.R., et al., *Ensembl 2018.* Nucleic Acids Res, 2018. **46**(D1): p. D754-D761.

8. ENSEMBL, *Gallus gallus 5.0 86 GTF*. 2016: [www.ensembl.org](file:///D:\Dropbox\Abasht_Lab_Folder_Joe_Tomlinson\ASE_PROJECT_EVERYTHING\VADT_PAPER_ALL_DOCUMENTS\FINAL_Paper_Submission\Supplemental_Sections\www.ensembl.org).

9. Broad. *Picard*. 2016; Available from: <https://broadinstitute.github.io/picard/>.

10. Quinlan, A.R. and I.M. Hall, *BEDTools: a flexible suite of utilities for comparing genomic features.* Bioinformatics, 2010. **26**(6): p. 841-2.

11. Wang, L., S. Wang, and W. Li, *RSeQC: quality control of RNA-seq experiments.* Bioinformatics, 2012. **28**(16): p. 2184-5.

12. ENSEMBL, *Gallus gallus 6.0 98 GTF*. 2019: [www.ensembl.org](file:///D:\Dropbox\Abasht_Lab_Folder_Joe_Tomlinson\ASE_PROJECT_EVERYTHING\VADT_PAPER_ALL_DOCUMENTS\FINAL_Paper_Submission\Supplemental_Sections\www.ensembl.org).

13. Sun, Y., et al., *Pseudogenes as weaknesses of ACTB (Actb) and GAPDH (Gapdh) used as reference genes in reverse transcription and polymerase chain reactions.* PLoS One, 2012. **7**(8): p. e41659.

14. Wells, L., K.A. Edwards, and S.I. Bernstein, *Myosin heavy chain isoforms regulate muscle function but not myofibril assembly.* EMBO, 1996. **15**(17): p. 4454-4459.

15. ENSEMBL, *Genome Assembly: Gallus_gallus-6.0*. 2019: [www.ensemble.org](file:///D:\Dropbox\Abasht_Lab_Folder_Joe_Tomlinson\ASE_PROJECT_EVERYTHING\VADT_PAPER_ALL_DOCUMENTS\FINAL_Paper_Submission\Supplemental_Sections\www.ensemble.org).

16. McLaren, W., et al., *The Ensembl Variant Effect Predictor.* Genome Biol, 2016. **17**(1): p. 122.

17. Fisher, R., *Statistical Methods for Research Workers (Thirteenth Edition-Revised)*. 1958, New York: Hafner Publishing Company Inc.

18. Benjamini, Y. and Y. Hochberg, *Controlling the false discovery rate: a pratical and powerful approach to multiple testing.* Journal of the Royal Statistical Society, 1995. **57**(1): p. 289-300.

19. Huang da, W., B.T. Sherman, and R.A. Lempicki, *Systematic and integrative analysis of large gene lists using DAVID bioinformatics resources.* Nat Protoc, 2009. **4**(1): p. 44-57.

20. Huang da, W., B.T. Sherman, and R.A. Lempicki, *Bioinformatics enrichment tools: paths toward the comprehensive functional analysis of large gene lists.* Nucleic Acids Res, 2009. **37**(1): p. 1-13.

21. Jones, E., et al. *SciPy (the library)*. 2001- 12-03-2017]; Available from: <http://www.scipy.org/>.
