# Supplementary material for: Investigation of allele specific expression in various tissues of broiler chickens using the detection tool VADT
Source: Sci Rep. 2021 Feb 17;11:3968. doi: 10.1038/s41598-021-83459-8 (PMC7889858; doi:10.1038/s41598-021-83459-8)
Supplement: Supplementary file 2 — Supplementary Information 2. [file 41598_2021_83459_MOESM2_ESM.docx]

**Investigation of Allele Specific Expression in Various Tissues of Broiler Chickens Using the Detection Tool VADT**

**M. Joseph Tomlinson IV ^§1, 5^, Shawn W. Polson^2,3,5^, Jing Qiu^4,5^, Juniper A. Lake^1,5^, William Lee^,6^, Behnam Abasht*^1,5^**

**Affiliations**

1. Department of Animal and Food Sciences, University of Delaware

2. Department of Computer and Information Sciences, University of Delaware

3. Department of Biological Sciences, University of Delaware

4. Department of Applied Economics and Statistics, University of Delaware

5. Center for Bioinformatics and Computational Biology, University of Delaware

6. Maple Leaf Farms, Inc. Leesburg, IN 46538, USA

**^§^** First Author

* Corresponding author: abasht@udel.edu

1. Department of Animal and Food Sciences, University of Delaware, 531 South College Ave, Newark, DE 19716, USA

**Supplemental Section 2**

**Adjusting p-values for Multidimensional Data to Control Mixed Directional FDR**

In the field of genetics there has been an explosion of extremely large multi-dimensional datasets like those derived from microarrays corresponding to gene expression or other similar type datasets, where for example genes are found on the y-axis and samples across the x-axis and each instance assigned a p-value derived from a statistical test. For such situation, the multiple testing problem is two dimensional: there are multiple tests for each variant and there are multiple variants. Guo *et al* (2010) [1] proposed a method to perform multi-dimensional correction of p-values to control for the mdFDR. The mdFDR is an error rate that combines both type I error (false positive) and directional errors (a gene is declared to be up regulated when it is down-regulated or vice versa) in the FDR framework.

Briefly speaking, Guo *et al*’s procedure apply Bonferroni correction to the multiple tests (or samples) for each variant, which results in Bonferroni pooled p-values for each variant. Then it applies Benjamini and Hochberg’s FDR controlling procedure [2] to the Bonferroni pooled p-values of all variants and detect R significant variants. For each of the R significant variants, the original p-value of each test (sample) is compared to a cutoff value, $\alpha R/qm$, where q is the number of the tests for each variant, m is the total number of variants, R is the number of significant variants and $\alpha$ is the targeted error rate. The significant individual test or sample is declared to be either positive or negative depending on the sign of the test statistics where the null hypothesis is the parameter of interest is zero. Such procedure will control mdFDR at level $\alpha$. The formal definition of mdFDR is

$$mdFDR=E\{\frac{V+S}{max\left( R,1 \right)}\}=E\{\frac{V}{max\left( R,1 \right)}\}+E\{\frac{S}{max\left( R,1 \right)}\}=FDR+dFDR,$$

where *V* represents the number of significant variants which are not ASE in any of the samples, *S* is the number of variants that are truly ASE in at least one of the samples but at least one directional mistake has been made while deciding upon the signs of the parameters and dFDR is the pure directional FDR. Note that mdFDR is the sum of the overall FDR(OFDR) and the pure directional FDR and hence controlling mdFDR is a stronger control of errors than the OFDR .

This method considers the entire dataset when controlling for type 1 error and error introduced by directionality and can be summarized in three major steps as outlined below [1]. We specifically implemented this procedure on a multi-dimensional dataset where variants are on the y-axis and p-values go across the x-axis for various samples as seen in Table 1. Each step is carefully broken down for simplicity and accuracy. In the demonstration below, we made two minor adjustments to Guo *et al*’s procedure: (1) we allow the number of tests to vary for each variant; (2) we did not make directional decisions at the end. The modified procedure is the procedure 2 proposed by Li and Ghosh (2014) [3], where they show such procedure controls the overall FDR at the variant level if no directional decision is made. According to Li and Ghosh (2014) [3], one can improve the power of Guo *et al*’s procedure by using Holm’s procedure (1979) [4] in the first step of obtaining the pooled p-values. However, for simplicity, we use the Bonferroni’s pooled p-value in the demonstration below.

**Table 1.** Example multidimensional data, where y-axis corresponds to a specific variants and x-axis with samples with corresponding p-values from a prior statistical test

$$P_{\mathrm{ij}}$$

q_i_

$$(P_{ij}{)q}_{i}$$

|  | Sample_1 | Sample_2 | Sample_3 |
| --- | --- | --- | --- |
| Variant_1 | 0.003 | 0.01 | 0.03 |
| Variant_2 | 0.01 | 0.0011 | 0.2 |
| Variant_3 | 0.001 | 0.05 | 0.07 |
| Variant 4 | 0.0035 | NaN | 0.5 |

**Step 1.** Perform a Bonferroni Correction on the lowest p-value of each variant to obtain a “Pooled” p-value.

$$(\min_{j} P_{ij}) q\_i$$

q_i_

$$(P_{ij}{)q}_{i}$$

Take lowest p-values obtained for each variant and multiply by the number of tests for Bonferroni Correction

number of tests

|  |  | q_i_ |  | Equation |  | Bonferroni pooled p-value |
| --- | --- | --- | --- | --- | --- | --- |
| Variant_1 |  | 3 |  | 0.003 x 3 |  | 0.009 |
| Variant_2 |  | 3 |  | 0.0011 x 3 |  | 0.0033 |
| Variant_3 |  | 3 |  | 0.001 x 3 |  | 0.003 |
| Variant 4 |  | 2 |  | .0035 x 2 |  | 0.007 |

**Step 2.** Adjustment of the Bonferroni pooled p-values using Benjamini Hochberg method [2] and determine how many values pass the 0.05 statistical significance threshold. The steps of the adjustment are outlined below. The final adjusted p-values were then compared for significance (<0.05) where it was determined all four variants passed.

Equation for one instance, overall process is iterative as shown below

$$Calc. adjustment= \frac{pooled p_{value_{i}}*No.Variants}{pooled p\_value\_Rank}$$

**Process:**

**1)** The Bonferroni pooled p-values are sorted largest to smallest.

**2)** Largest Bonferroni pooled p-values value is corrected using above the equation and this value becomes the Benjamini-Hochberg (BH) adjusted p-value.

**3)** The following p-value in the sorted list is corrected using the same equation but is compared to the prior adjusted p-valu**e.** If the currently adjusted p-value is larger than the prior adjusted p-value in the list, it is replaced with the prior value (cases in **bold** in table below), otherwise the calculated value becomes the adjusted value.

**4)** Iteratively repeat process moving down the list.

| Rank |  | Calc Adj Value |  | Determine Min |  | BH Adj p-value |
| --- | --- | --- | --- | --- | --- | --- |
| 4 |  | 0.009 x 4 / 4 = 0.009 |  | min(0.009) |  | 0.0090 |
| 3 |  | 0.007 x 4 / 3 = 0.0093 |  | min(**0.009**, 0.0093) |  | 0.0090 |
| 2 |  | 0.0033 x 4 / 2 = 0.0066 |  | min(0.0066, 0.009) |  | 0.0066 |
| 1 |  | 0.003 x 4 / 1 = 0.012 |  | min(**0.0066**, 0.012) |  | 0.0066 |

**Compare to 0.05**

Total number of variants that pass

in this example is 4

**Step 3.** Determine the adjusted cutoff values for the original p-values of each significant variant using the following equation and determine which tests (or samples) of the variant is significant. It is important to note the cutoff value needs to be re-calculated for each variant if the number of possible tests $q_{i}$changes.

The Number of Significant Variants Based on the BH Adjustment.

0.05

$$\tilde{P}_{\mathrm{ij}}\leq\frac{R* \alpha}{q_{i}*m}$$

Total Number of Variants Analyzed

Number of Possible Tests

$$\tilde{P}_{\mathrm{ij}}\leq\frac{R* \alpha}{q*m}= \frac{4*0.05}{3*4}=0.017$$

Variant 1

Determine Which Samples are Less Than This Adjusted Threshold

**Variant 1 Results**

|  | Sample_1 | Sample_2 | Sample_3 |
| --- | --- | --- | --- |
| Variant_1 | 0.003 | 0.01 | 0.03 |
|  |  |  |  |
| **Verdict** | pass | pass | fail |

**Final Results.** Below is shown the analysis of the entire dataset showing which p-values pass the newly defined significance threshold, shown on the right. The first three variants have the same defined threshold of 0.017 and the fourth variant which is has one less value, due to a NaN, has a threshold of 0.025. Each threshold for significance was calculated based on the prior outlined steps. Specifically, for each sample if it is considered statistically significant it is marked as pass and not significant as fail followed by its original p-value.

**Examining the Entire Table of Results**

|  | Sample_1 | Sample_2 | Sample_3 |
| --- | --- | --- | --- |
| Variant_1 | pass\|0.003 | pass\|0.01 | fail\|0.03 |
| Variant_2 | pass\|0.01 | pass\|0.0011 | fail\|0.2 |
| Variant_3 | pass\|0.001 | fail\|0.05 | fail\|0.07 |
| Variant_4 | pass\|0.0035 | nan | fail\|0.5 |

Threshold = 0.017

Threshold = 0.025

Work Cited

1. Guo, W., S.K. Sarkar, and S.D. Peddada, *Controlling false discoveries in multidimensional directional decisions, with applications to gene expression data on ordered categories.* Biometrics, 2010. **66**(2): p. 485-92.

2. Benjamini, Y. and Y. Hochberg, *Controlling the false discovery rate: a pratical and powerful approach to multiple testing.* Journal of the Royal Statistical Society, 1995. **57**(1): p. 289-300.

3. Li, Y. and D. Ghosh, *A two-step hierarchical hypothesis set testing framework, with applications to gene expression data on ordered categories.* BMC Bioinformatics, 2014. **15**(108): p. 1-11.

4. Holm, S., *A simple sequentially rejective multiple test procedure.* Scand J Statist, 1979. **6**(2): p. 65-70.
